# Framework for personalized prediction of treatment response in relapsing remitting multiple sclerosis

**DOI:** 10.1186/s12874-020-0906-6

**Published:** 2020-02-07

**Authors:** E. Stühler, S. Braune, F. Lionetto, Y. Heer, E. Jules, C. Westermann, A. Bergmann, P. van Hövell, NeuroTransData Study Group

**Affiliations:** 1 PwC Digital Services, Zürich, Switzerland; 2 NeuroTransData, Neuburg an der Donau, Germany

**Keywords:** Relapsing remitting multiple sclerosis, Personalized medicine, Clinical decision support, Personalized health record, Personalized predictive models, Bayesian generalized linear model

## Abstract

**Background:**

Personalized healthcare promises to successfully advance the treatment of heterogeneous neurological disorders such as relapsing remitting multiple sclerosis by addressing the caveats of traditional healthcare. This study presents a framework for personalized prediction of treatment response based on real-world data from the NeuroTransData network.

**Methods:**

A framework for personalized prediction of response to various treatments currently available for relapsing remitting multiple sclerosis patients was proposed. Two indicators of therapy effectiveness were used: number of relapses, and confirmed disability progression. The following steps were performed: (1) Data preprocessing and selection of predictors according to quality and inclusion criteria; (2) Implementation of hierarchical Bayesian generalized linear models for estimating treatment response; (3) Validation of the resulting predictive models based on several performance measures and routines, together with additional analyses that focus on evaluating the usability in clinical practice, such as comparing predicted treatment response with the empirically observed course of multiple sclerosis for different adherence profiles.

**Results:**

The results revealed that the predictive models provide robust and accurate predictions and generalize to new patients and clinical sites. Three different out-of-sample validation schemes (10-fold cross-validation, leave-one-site-out cross-validation, and excluding a test set) were employed to assess generalizability based on three different statistical performance measures (mean squared error, Harrell’s concordance statistic, and negative log-likelihood). Sensitivity to different choices of the priors, to the characteristics of the underlying patient population, and to the sample size, was assessed. Finally, it was shown that model predictions are clinically meaningful.

**Conclusions:**

Applying personalized predictive models in relapsing remitting multiple sclerosis patients is still new territory that is rapidly evolving and has many challenges. The proposed framework addresses the following challenges: robustness and accuracy of the predictions, generalizability to new patients and clinical sites and comparability of the predicted effectiveness of different therapies. The methodological and clinical soundness of the results builds the basis for a future support of patients and doctors when the current treatment is not generating the desired effect and they are considering a therapy switch.

**Graphical abstract:**

(A) The framework is developed using quality-proven real-world data of patients with relapsing remitting multiple sclerosis. Patients have heterogeneous individual characteristics and diverse disease profiles, indicated for example by variations in frequency of relapses and degree of disability. Longitudinal characteristics regarding disease history (e.g. number of previous relapses in the last 12 months) are extracted at the time of an intended therapy switch, i.e. at time point “Today” (left). All clinical parameters are captured in a standardized way (right). (B) The model predicts the course of the disease based on the observed data (panel A), and is able to account for the impact of various available therapies on chosen clinical endpoints. The resulting ranking of therapies has a dependency on patient characteristics, illustrated here by a different highest ranked therapy depending on the number of relapse in the previous 12 months. (C) The model is evaluated for various generalization properties. Compared to performance on the training set (gray) it is able to predict for new patients not part of the training set (red).Top: Prediction for new patients. Middle: Prediction for new clinical sites. Bottom: Prediction for different time windows. (D) In order to assess the clinical impact of the model, disease activity is compared between patients treated with the highest ranked therapy and those treated with any of the other therapies. Patients adhering to the highest ranked therapy are associated with a better disease outcome when compared to those who did not. 
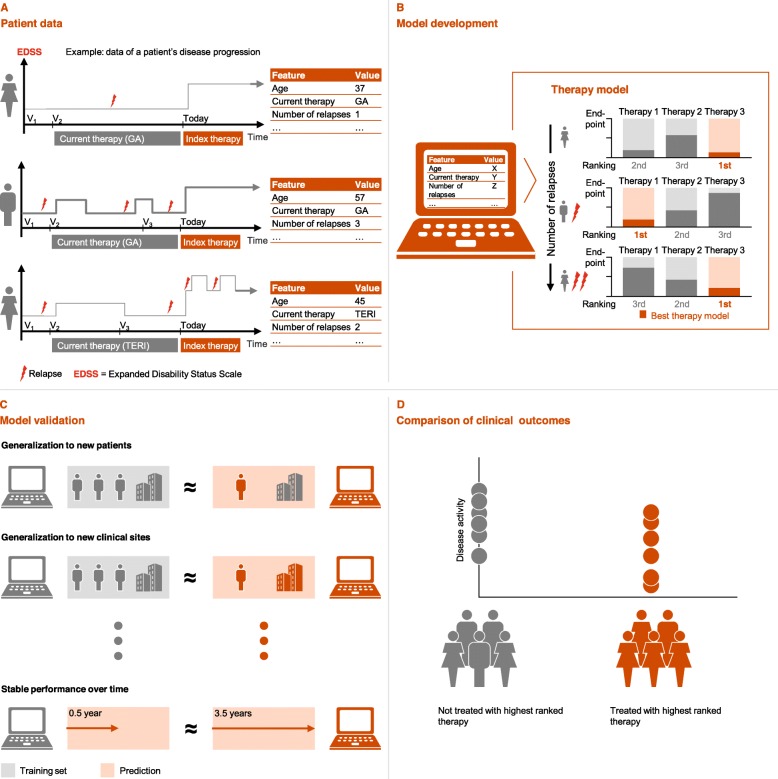

## Background

Knowledge about the effectiveness of available treatments is typically based on results from randomized controlled trials (RCTs). However, these results are derived in a controlled, constrained setting and do not necessarily reflect real-world patient populations and drug labels. In addition, results from RCTs are based on group-level differences, while treatment decisions require individual-level information to enable optimal treatment allocation for an individual patient. This can cause a ‘trial-and-error paradigm of treatment allocation’ [1], in which the individual patient undergoes several therapy switches until a suitable treatment is found. Real-world data are gaining increasing importance to fill this gap between RCTs and utilization of treatment options in daily practice. This is also why the European Medical Agency and the Food and Drug Administration in the USA are seeing the availability of real-world data as an emerging opportunity to improve treatment quality and allocation of resources [2] [3] [4] [5] [6]. In the field of multiple sclerosis (MS), several registries have captured qualified clinical data for more than 10 years, including MSBase (founded in 2004, multinational), OFSEP (2001, France), Swedish MS registry (2001, Sweden), MSDS3D (2010, Germany), and NeuroTransData (NTD; 2008, Germany).

MS is the most prevalent neurological auto-immune disorder of the central nervous system and affects patients in the most dynamic and productive time of their lives by causing severe physical disability and mental handicap, thus impairing abilities for social and professional participation over time in the majority of those affected. The treatment landscape of MS is continually changing: since 2000, more than 900 clinical studies have been listed in the clinical trials registry created by the National Institutes of Health alone. Following the first Interferon-β1b injectable therapy in 1995, 14 different disease modifying therapies (DMTs) based on eight approved compounds became available in the EU in 2017 for the relapsing remitting form of multiple sclerosis (RRMS) [2].

Disease mechanisms and course of RRMS are heterogeneous and challenging to predict at a group level, and even more in an individual patient. This heterogeneity, the impact of clinical outcomes on quality of life and the large number of treatment options make a personalized approach for a tailored treatment of patients desirable.

This study is built on previous research on personalized medicine [5–10]. It contributes to an ongoing advancement on the personalization of RRMS treatment allocation by proposing a framework for comparing the effectiveness of DMTs at patient level. Two indicators of treatment effectiveness are taken into account: the number of on-therapy relapses experienced by the patient, and the occurrence of a confirmed disability progression (CDP) during the therapy. The statistical approach relies on hierarchical Bayesian generalized linear models (GLMs).

This study is based on the NTD MS registry, where physicians in Germany capture quality-proven real-world data in a large number of different clinical sites and for heterogeneous patients and disease histories. This provides the basis for the generalization of the predictive models to a wide range of patients and clinical sites that were not part of the model development. The methodological and clinical soundness of the results of this framework is thoroughly evaluated in addition by comparing the predicted treatment response with the clinically observed course of MS for different adherence profiles.

Applying personalized predictive models in RRMS patients is still a new territory that is rapidly evolving and has many challenges. The objective of this study is to address some of these challenges by providing robust and reliable predictions of treatment response based on real-world data. This builds the basis for a future support of patients and doctors when the ongoing treatment fails and a therapy switch is considered.

## Methods

This study proposes a framework for personalized prediction of effectiveness of various therapies currently available for RRMS patients. The framework comprises the following steps: (i) data preprocessing, selection of predictors according to quality and inclusion criteria, and definition of two indicators of therapy effectiveness (Section 2.1); (ii) model development using hierarchical Bayesian GLMs for estimating therapy response according to both indicators of therapy effectiveness (Section 2.2); (iii) model performance assessment based on state-of-the-art performance measures and procedures, together with additional analyses that focus on applicability in clinical practice, i.e. on generalizability to new data and on comparability of predictions for different treatments (Section 2.3).

### Data

This study employed clinical real-world data recorded in the NTD MS registry. NTD is a Germany-wide network of physicians in the fields of neurology and psychiatry that was founded in 2008. Currently, 153 neurologists in 78 offices work in NTD practices serving about 600,000 outpatients per year. Each practice is certified according to network-specific and ISO 9001 criteria. Compliance with these criteria is audited annually by an external certified audit organization. The NTD MS registry includes about 25,000 patients with MS, which represents about 15% of all MS patients in Germany. In this database, demographic and clinical parameters are captured in real time over an average of 3.7 visits and Expanded Disability Status Scale (EDSS) assessments per year per patient. Standardized clinical assessments of functional system scores and EDSS calculation are performed by certified raters (http://www.neurostatus.net). All personnel undergoes regular training to ensure quality of data in the database. This quality is monitored by the NTD data management team. Data input is checked for inconsistencies and errors by also using an error analysis program. Both automatic and manually executed queries are implemented to further ensure data quality, e.g. checks for inconsistencies and requests for missing information. All data are pseudonymized and pooled to form the NTD MS database. The codes uniquely identifying patients are managed by the Institute for medical information processing, Biometry and Epidemiology (Institut für medizinische Informationsverarbeitung, Biometrie und Epidemiologie (IBE)) at the Ludwig Maximilian University in Munich, Germany, acting as an external trust center. The data acquisition protocol described above was approved by the ethical committee of the Bavarian Medical Board (Bayerische Landesärztekammer; June 14, 2012) and re-approved by the ethical committee of the Medical Board North-Rhine (Ärztekammer Nordrhein; April 25, 2017).

For this study, data were extracted from the NTD MS database on July 1, 2018.

#### Predictors

The predictors that were used to model therapy effectiveness are listed in Table 1. An overview of their distribution and discretization is provided in Additional file 1: Table S1.3. All predictors were defined and selected based on prior scientific research and clinical expertise (SB, AB) [11].

**Table 1:** List of model predictors, along with code names for shorter reference across the study

Code name	Description
Age	Age at the start of the index therapy
Gender	Gender
EDSS	EDSS (measured at most 6 months before or 3 months after the start of the therapy cycle, and at least 84 days after a relapse)
Index / Index therapy	DMT taken during the therapy cycle
Current / Current therapy	DMT taken prior to the start of the therapy cycle
Diagnosis distance	Time elapsed between MS diagnosis and start of index therapy
Relapse distance	Time elapsed between the last relapse preceding the start of the index therapy and the start of the index therapy
Relapses count	Number of relapses in the year prior to the start of the index therapy
DMTs count	Number of DMTs taken prior to the start of the index therapy
Second-line	Whether a second-line DMT has been taken before the start of the index therapy
Current duration	Duration of the current therapy
Index duration	Duration of the index therapy
Clinical site	Clinical site where the course of MS is observed

*EDSS* expanded disability status scale, *DMT* disease modifying therapy, *Second-line* DMT to be employed by label of the European Medical Agency if previous DMT failed to achieve sufficient control of disease activity (of the DMTs considered in this work, this applies to Fingolimod and Natalizumab), *MS* multiple sclerosis.

#### Data quality and inclusion criteria

The data used for model development consist of therapy cycles, i.e. each observation corresponds to a therapy cycle. Several quality and inclusion criteria were applied for data preprocessing and patient population selection, respectively.

The selected observations fulfilled quality criteria related to validity, accuracy, completeness, consistency and uniformity of the information in the database, including: all predictors were available at the start of the index therapy, at least one relapse was documented before the start of the index therapy, patients were required to have at least one documented EDSS measurement before the start of the index therapy. Extreme therapy cycles with annualized relapse rate above 12 per year were excluded from the study.

The following inclusion criteria were applied: patients were required to be at least 18 years old, EDSS before the start of the index therapy was required to be less than or equal to six, index therapy was required to be one of the following: Dimethylfumarat (DMF), Fingolimod (FTY), Glatirameracetat (GA), Interferon-ß1 (IF), Natalizumab (NA) or Teriflunomide (TERI). Therapies that were prescribed within 6 months of MS diagnosis without a previous treatment failure were excluded as they do not represent therapy switches during the course of RRMS. If more than one therapy cycle was available for a single patient, one therapy was randomly selected, while the others were discarded. Therapy cycles corresponding to clinical sites with only one remaining patient were also excluded.

After the quality and inclusion criteria were applied, 90% of the therapy cycles (3119) were used for model development and validation, and 10% of the therapy cycles (314) were used as test set for validation as described below in Section 2.3.3.

A detailed overview of the data selection process is shown in Additional file 1: Table S1.1. A comparison of the responses of interest and of the predictors before and after the quality and inclusion criteria were applied is presented in Additional file 1: Tables S1.2, S1.3 and Figure S1.3.

#### Indicators of therapy effectiveness

Number of relapses and confirmed disability progression (CDP) during the observation time of a therapy cycle are established clinical indicators for therapy effectiveness in RRMS [7, 8]. Both indicators were therefore used to measure the effectiveness of index therapies. Additionally, probabilities of being free of relapse and free of CDP, respectively, were derived for validation [9].

A CDP was defined as a worsening of at least 1.0 point when the previous EDSS is 5.5 or lower and 0.5 point otherwise; the worsening must be sustained for at least 3  months and must be confirmed by at least one other valid EDSS measurement. A more detailed definition of CDP is included in Additional file 2.

### Model development

The number of on-therapy relapses was modelled as following a negative binomial distribution whose mean and shape parameters depend on individual patient characteristics and index therapy. The occurrence of a CDP was modelled as following a binomial distribution, where the probability of observing a CDP depends on individual patient characteristics and index therapy.

In both cases, a hierarchical Bayesian GLM was employed [12]. The correlation that typically arises between measurements coming from the same clinical site was addressed by modeling a random intercept. The duration of each observed therapy cycle was incorporated in the models as an offset term, since the number of relapses and the probability of observing a CDP is expected to be larger for longer exposure, i.e. observation time of index therapy. A detailed description of the models is presented in Additional file 3.

In this study, Bayesian estimation was used due to the advantages offered by the possibility to incorporate prior information, which also allows for regularization [13]. The specific values that were given to the parameters’ priors are summarized in Table 2. These priors are weakly informative, in line with the values proposed by [14]. The parameters are assumed to be independent.

**Table 2:** Default priors assigned to the relapse and CDP models’ parameters

Model	Intercept	Fixed effects	Dispersion	Standard deviation of random intercepts
Relapse	N(0, 10)	N(0, 2.5)	Half-Cauchy(0, 5)	Gamma(1, 1)
CDP	N(0, 10)	N(0, 2.5)	–	Gamma(1, 1)

*CDP* confirmed disability progression.

Models were fitted with version 2.14.1 of the *rstanarm* package in R [13]. This implementation uses the Hamiltonian Monte Carlo approach to draw samples from the parameters’ posterior joint distribution. For each Markov chain started to this purpose, the convergence to the target distribution was assessed using the Gelman and Rubin potential scale reduction statistic $\hat{R}$ [13]. For each of the samples and for each of the six considered index therapies (Section 2.1.2), the number of relapses or the occurrence of a CDP were predicted for each observation by disregarding clinic-specific random effects, i.e. by setting all random intercepts to zero (in *rstanarm*, this is done by setting the ‘re.form’ argument of the posterior_predict function to ‘~ 0′). A new patient will thereby have consistent predictions across different clinical sites. These predictions obtained from the posterior distribution were summarized by looking (i) at their average and at the fraction of those that predict an absence of relapse (relapse model), and (ii) at the fraction of those that predict an absence of CDP (CDP model).

### Model performance assessment

In this section, the following content is presented: model calibration (Section 2.3.1), statistical measures of model performance (Section 2.3.2.), model generalizability (Section 2.3.3), comparison with nested models of lower complexity (Section 2.3.4), sensitivity of the models to different choices of the priors, to the characteristics of the patient population, and to the sample size (Section 2.3.5), and comparison of treatment effectiveness predicted by the models (Section 2.3.6).

#### Model calibration

The agreement between the predicted and observed outcomes was assessed by distributing the therapy cycles into several bins of predicted outcomes. The bin size was chosen such that there are 20 equally-populated bins in total, covering the full range of the predicted outcomes. For each bin, the mean predicted outcome was compared with the mean observed outcome. If the model is well-calibrated, the two quantities are expected to be close to each other. The agreement between predictions and observations was studied for all therapy cycles and also for each DMT separately. The adoption of equally-populated bins rather than equally-sized bins has the advantage that the statistical uncertainty due to the population size of each bin is the same for all points in the calibration plot.

#### Statistical measures of model performance

Model performance was evaluated via mean squared error (MSE), negative log-likelihood and Harrell’s concordance statistic (C-Index).

The C-Index was used to analyze the ability of the models to discriminate among different responses, in this case to discriminate between none and at least one relapse, and between the occurrence and absence of a CDP, respectively. When comparing predicted and observed indicators of therapy effectiveness, therapy cycles with roughly the same duration were matched. This is achieved by allowing for up to 6 months difference if the smaller of the two durations is less than half a year, and up to 12 months difference otherwise [15].

The negative log-likelihood per patient was obtained following the approach in [12], page 169, and using the *log_lik* function of the *rstanarm* package [13]. The negative log-likelihood for the full patient population was obtained by summing the negative log-likelihoods per patient.

Although the models allow to make predictions for the effectiveness of all six therapies included in this study (Section 2.1.2), statistical measures were only evaluated where the associated indicator of therapy effectiveness could be observed, i.e. using the predictions for the observed index therapy.

#### Model generalizability

The generalizability of the models was assessed using three different out-of-sample validation schemes. The first validation scheme consisted in evaluating the model performance using a 10-fold cross-validation. The second validation scheme used a leave-one-site-out cross-validation with respect to the clinical site. Patients from the same clinical site were excluded from the sample that was used to fit the model and then used to test how well this model performs. The procedure was repeated for each clinical site. The third validation scheme evaluated the model performance on the test set.

Performance measures were calculated using out-of-sample predictions as well as in-sample predictions. Each in-sample prediction was obtained from one randomly selected training fold. Therefore, exactly one out-of-sample and one in-sample prediction per therapy cycle were retained. Out-of-sample and in-sample performance measures were compared in order to identify overfitting. The robustness of the performance measures was assessed by repeating the 10-fold cross-validation 40 times, which allowed to compute standard errors.

The modeling approach described in Section 2.2 leads to the generation of six predictions per patient, one for each of the six therapies under consideration. Only predictions for the observed index therapy were retained when analyzing generalizability.

#### Comparison with nested models

The models presented above allow to make comparable predictions for all six therapies included in this study for each patient and each indicator of therapy effectiveness. The impact of the patient characteristics and their interactions with the index therapy on the model predictions was evaluated by comparing the model of Section 2.2 with two models of lower complexity. These two models were nested in the predictive model.

The first nested model, referred to as **non-personalized model**, does not have a dependency on the patient characteristics (Table 3). This model returns a fixed ranking of the six therapies under consideration. The model is not personalized, since two patients with a therapy cycle of the same duration but different characteristics will obtain the same predicted response, and hence will have the same comparative therapy effectiveness profile.

**Table 3:** Overview of the predictors used for predictive models and nested models

	Non-personalized model	Prognostic model	Predictive model
Clinical site	x	x	x
Index therapy	x	x	x
Index duration	x	x	x
Age		x	x
Gender		x	x
EDSS		x	x
Second-line		x	x
Current therapy		x	x
Current duration		x	x
Interaction (Current therapy, Current duration)		x	x
Diagnosis distance		x	x
Relapse distance		x	x
Relapses count		x	x
DMTs count		x	x
Interaction (Index therapy, Diagnosis distance)			x
Interaction (Index therapy, Gender)			x
Interaction (Index therapy, Relapses count)			x
Interaction (Index therapy, Second-line)			x

*EDSS* expanded disability status scale, *DMT* disease modifying therapy, *Second-line* DMT to be employed by label of the European Medical Agency if previous DMT failed to achieve sufficient control of disease activity.

The second nested model, referred to as **prognostic model**, has a dependency on the patient characteristics but not on their interactions with the index therapy (Table 3).

This model is an extension of the non-personalized model that additionally allows for personalization, that is, for patient characteristics to have an impact on the predicted response. However, it is important to note here that patient characteristics are not allowed to interact with the index therapy, i.e. there is no personalization in the obtained ranking of the six therapies under consideration.

The predictive model in this study differs from the prognostic model by the addition that individual patient characteristics were allowed to interact with the therapy, i.e. the therapy effectiveness and corresponding ranking were allowed to differ for different patients.

#### Sensitivity analysis

The predictions’ robustness was tested with respect to different choices of the priors, to the characteristics of the underlying patient population, and to sample size. Methods and results are presented in detail in Additional file 5, Additional file 6 and Additional file 7.

#### Comparison of predicted therapy response

The modeling approach presented above leads to the generation of six predictions per patient (Section 2.2), one for each of the six therapies under consideration. As both predictive models allow therapy effectiveness to differ for different patient characteristics, a personalized ranking of therapies was obtained for each patient. Note that this ranking only applies with respect to the chosen indicator of therapy effectiveness, i.e. in this case either the lowest predicted number of relapses or the lowest predicted probability of observing a CDP, and does not represent an overall therapy recommendation which would account for multiple determinants. In order to evaluate the usability of the models in clinical practice, average observed treatment responses were compared between patients who received the highest ranked therapy (denoted as DMT^*^ in the following) and those who did not.

To avoid potential confounders on responses, the distribution of each predictor was matched between patients who received DMT* and those who did not with a propensity-score-based weighting as implemented by the *twang* package [16, 17]. The propensity-score-based weighting allowed to match the distributions of the covariates of the two groups without having to discard any data [16, 18, 19]. It was implemented based on age, relapses count (in the past 12 months), EDSS as categorized in [8], and diagnosis distance. The distributions of the covariates of each group were matched by using the population weights to estimate the average treatment effect of the population [16, 17], while all other settings were kept to the default settings of the *twang* package [16, 17].

To test the statistical significance of the group differences between patients receiving DMT* and those who did not, a weighted GLM was employed according to an analysis of outcomes approach [16]. *P*-values were derived from the GLM based on the estimated significance of the relevant intercept and slope coefficients. For each observation, an indicator variable was used to specify whether the patient received DMT*. A negative binomial GLM was used for the observed number of relapses [18] and a binomial GLM was employed for the observed occurrence of a CDP as follows:

$$ svyglm. nb\left( observed. number. relapses\sim took. DM{T}^{\ast }+ offset\left(\log \left( duration.{DMT}^{\ast}\right)\right), design= design. ps\right) $$

$$ svyglm\left( observed. occurence. CDP\sim took. DM{T}^{\ast }+ offset\left(\log \left( duration.{DMT}^{\ast}\right)\right), family= binomial, design= design. ps\right) $$

**Fig. 1:**
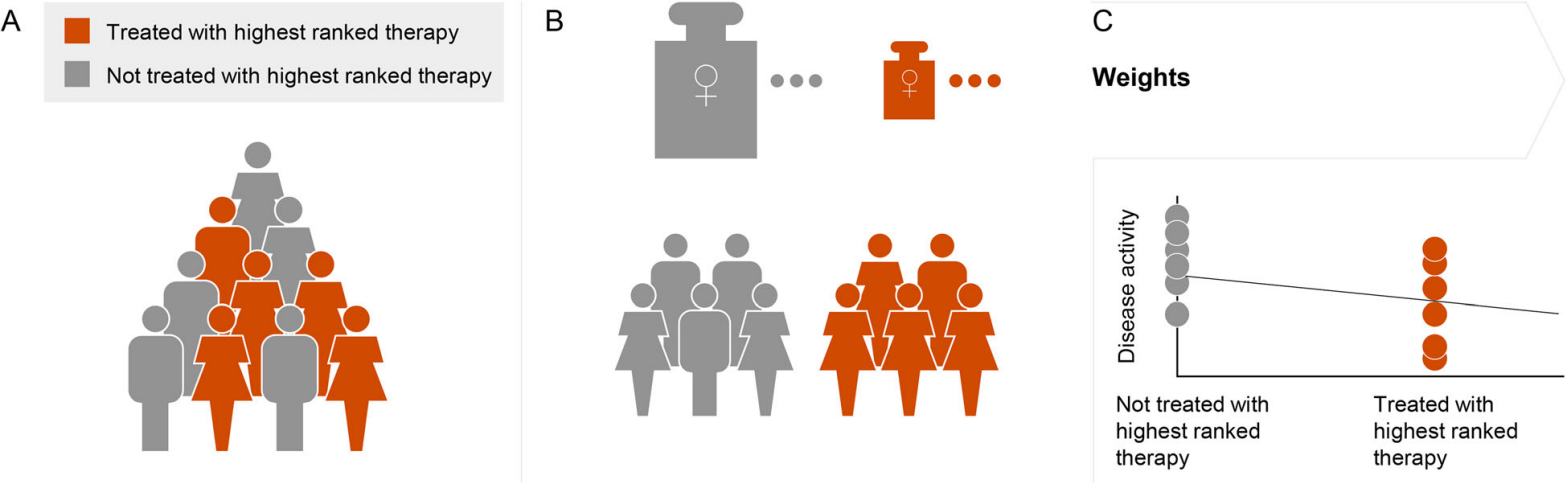
Comparison of predicted therapy. **a** Patients assigned to the same highest ranked therapy are divided into two groups: those who received indeed this therapy (red) and those who received another therapy (gray). **b** Weights are calculated for the two groups to mitigate the effect of confounds on the analysis. In particular, the weights are calculated such that each group matches the population statistics. As an example, this results in larger weights for females in the group with a smaller ratio of females compared with the overall occurrence than in the group with a larger ratio. **c** The weights are included in a survey-weighted GLM, where an indicator variable encodes membership to one of the two groups, and observation time is accounted for. The GLM allows for a propensity-score-based weighting of the clinically relevant outcomes of the two groups. The estimated slope allows then for a comparison of the disease activity between the two groups

The duration of each therapy cycle was included as offset to allow for cycles with heterogeneous observation time, i.e. to allow for the comparability of the results between patients with different index therapy durations. The procedure is illustrated in Fig. 1.

## Results

The following results are presented: overview of patient population after quality and inclusion criteria were applied (Section 3.1), importance of model coefficients (Section 3.2) and model performance assessment (Section 3.3).

### Patient population

After the inclusion criteria described in Section 2.1.2 have been applied, index therapy cycles consist of: Dimethylfumarat (22%), Fingolimod (25%), Glatirameracetat (13%), Interferon-ß1 (19%), Natalizumab (9%), and Teriflunomide (13%). At least 266 therapy cycles per DMT are available for model development. A detailed description of the patient population is reported in Additional file 1.

### Importance of model coefficients

The predictors associated to the eight largest fixed-effect parameter estimates (in magnitude) of the relapse and CDP models are reported in Table 4 and Table 5, respectively. The predictors are listed along with the signs and the posteriors’ median absolute deviations (MADs) of their corresponding estimates. Most of the ranked coefficients are interaction terms, two of which appear in both rankings: (i) the coefficient for the interaction between Natalizumab as index therapy and the diagnosis distance, and (ii) the coefficient for the interaction between Fingolimod as index therapy and the second-line therapy indicator. In the relapse model, the duration of the current therapy seems to have a significant impact both individually and when combined with Teriflunomide or Fingolimod. Note that the largest parameter estimate has the highest uncertainty. In the CDP model, having had a second-line DMT is particularly meaningful when the index therapy is Teriflunomide, Natalizumab or Fingolimod.

**Table 4:** Most important predictors in the relapse model

Rank^a^	Predictor	Sign^b^	MAD
1	Current = TERI: Current duration	–	4.301
2	Intercept	–	0.553
3	Current = TERI	–	1.406
4	Current = FTY: Current duration	+	1.983
5	Index = NA: Diagnosis distance	+	0.368
6	Current duration	+	1.414
7	Index = TERI: Relapses count	–	0.332
8	Index = FTY: Second-line = TRUE	–	0.335

*MAD* median absolute deviation, *TERI* Teriflunomide, *FTY* Fingolimod, *NA* Natalizumab, *Second-line* DMT to be employed by label of the European Medical Agency if previous DMT failed to achieve sufficient control of disease activity.

^a^ Ranked according to the magnitude of the median of the corresponding coefficient’s posterior distribution

^b^ A positive sign is associated with a boosting effect on the number of relapses; a negative sign is associated with a lessening effect on the number of relapses

**Table 5:** Most important predictors in the CDP model

Rank^a^	Predictor	Sign^b^	MAD
1	Intercept	–	0.721
2	Index = TERI: Second-line = TRUE	–	0.977
3	Index = NA: Second-line = TRUE	–	0.837
4	Index = NA: Diagnosis distance	+	0.518
5	Index = FTY: Second-line = TRUE	–	0.487
6	Current = IF: Current duration	–	1.604
7	Current = NA: Current duration	–	2.124
8	Current	+	0.711

*MAD* median absolute deviation, *TERI* Teriflunomide, *FTY* Fingolimod, *NA* Natalizumab, *IF* Interferon-ß1, *Second-line* DMT to be employed by label of the European Medical Agency if previous DMT failed to achieve sufficient control of disease activity.

^a^ Ranked according to the magnitude of the median of the corresponding coefficient’s posterior distribution

^b^ A positive sign is associated with a boosting effect on the likelihood of observing a CDP; a negative sign is associated with a lessening effect on the likelihood of observing a CDP

**Fig. 2:**
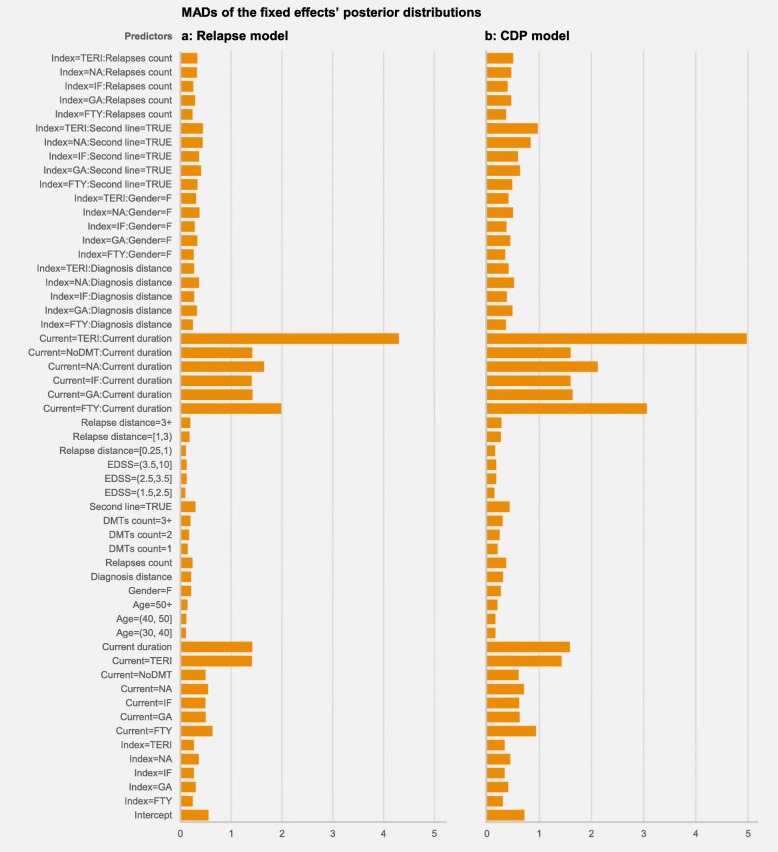
Model coefficients. MADs of the fixed effects’ posterior distributions in the relapse model (**a**) and in the CDP model (**b**)

Figure 2 displays the MADs of the fixed-effects’ posterior distributions in the relapse and the CDP models. In both cases, the estimates having highest uncertainty are those associated to the following predictors: duration of the current therapy, interaction between the current therapy and its duration, and Teriflunomide as a current therapy.

### Model performance assessment

Model performance was assessed via model calibration (Section 3.3.1), model generalizability (Section 3.3.2), comparison with nested models (Section 3.3.3), sensitivity analysis (Section 3.3.4) and comparison of observed therapy response to evaluate the usability of the models in clinical practice (Section 3.3.5).

#### Model calibration

The agreement between predictions and observations was studied for all DMTs and also for each DMT separately.

**Fig. 3:**
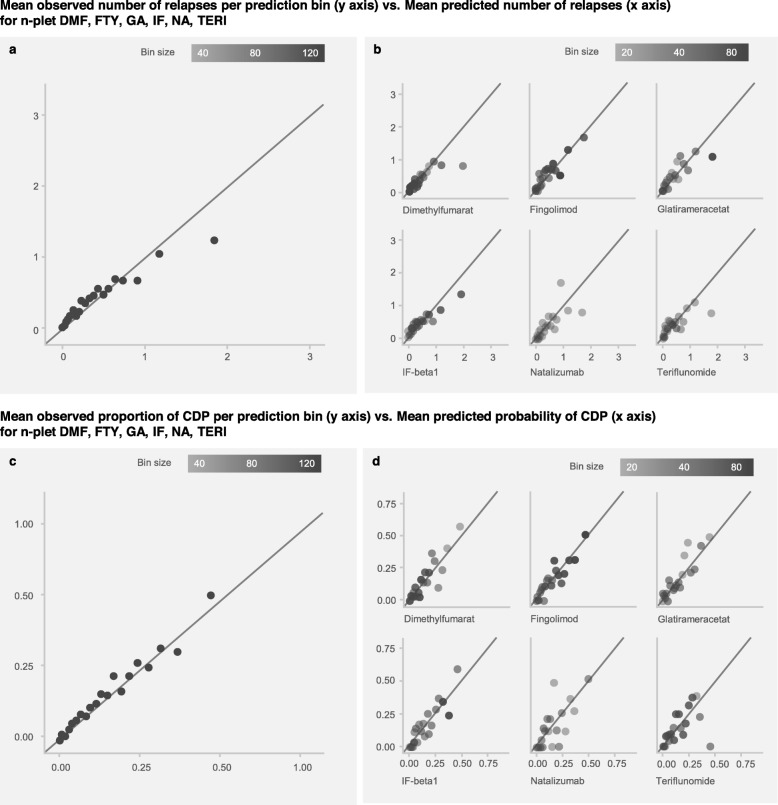
Calibration. Calibration of the relapse model (top) and CDP model (bottom) using equally-populated bins, when considering all DMTs together (left) and when considering each DMT separately (right). The predictions are split into 20 equally-populated bins from zero to four relapses, for the relapse model, and from zero to one, for the CDP model. Different shades of gray highlight a different population size

Both the relapse model and the CDP model are well calibrated for low values of the response, while the agreement between prediction and observation worsens for high response values (Fig. 3. As only few therapy cycles have a high response value, improvement can be expected as new data is collected.

The same calibration assessment was performed for each of the six considered index therapies the models are trained on. The resulting figures show the same trend as for the calibration with all DMTs (Fig. 3), indicating that the predictions for one selected index therapy do not systematically underestimate or overestimate the response when compared with the others.

This supports the conclusion that predictions for different therapies can be compared, and that the resulting therapy ranking is usable.

#### Model generalizability

The generalizability of the model was assessed using three different out-of-sample validation schemes (Section 2.3.3). All results are reported in Table 6, Table 7 and Table 8.

**Table 6:** Performance of predictive, prognostic and non-personalized models based on out-of-sample and in-sample predictions

Measure	Out-of-sample mean (SE)^a^	In-sample mean (SE)^a^	Sample size^b^	Response	Model
C-Index	0.5819 (0.0008)	0.6546 (0.0005)	307,784	CDP	predictive
C-Index	0.5625 (0.0007)	0.6220 (0.0004)	307,784	CDP	prognostic
C-Index	0.5467 (0.0006)	0.5649 (0.0005)	307,784	CDP	non-personalized
C-Index	0.6458 (0.0004)	0.6781 (0.0003)	505,724	relapse	predictive
C-Index	0.6482 (0.0003)	0.6700 (0.0002)	505,724	relapse	prognostic
C-Index	0.5531 (0.0003)	0.5609 (0.0003)	505,724	relapse	non-personalized
MSE	0.12497 (0.00005)	0.11928 (0.00004)	3119	CDP	predictive
MSE	0.12486 (0.00004)	0.12132 (0.00003)	3119	CDP	prognostic
MSE	0.12449 (0.00002)	0.12388 (0.00001)	3119	CDP	non-personalized
MSE	0.7554 (0.0008)	0.7097 (0.0006)	3119	relapse	predictive
MSE	0.7312 (0.0005)	0.7049 (0.0003)	3119	relapse	prognostic
MSE	0.7557 (0.0002)	0.7517 (0.0001)	3119	relapse	non-personalized
NLL	1252.6 (0.6)	1190.9 (0.4)	3119	CDP	predictive
NLL	1254.0 (0.5)	1215.5 (0.2)	3119	CDP	prognostic
NLL	1246.8 (0.2)	1240.3 (0.1)	3119	CDP	non-personalized
NLL	2580.8 (0.6)	2519.9 (0.4)	3119	relapse	predictive
NLL	2574.6 (0.5)	2534.9 (0.3)	3119	relapse	prognostic
NLL	2650.1 (0.2)	2641.9 (0.2)	3119	relapse	non-personalized

*SE* standard error of the mean, *CDP* confirmed disability progression, *C-Index* Harrell’s concordance statistic, *MSE* mean squared error, *NLL* negative log-likelihood.

^a^ Estimated by repeating 10-fold cross-validation 40 times

^b^ Refers either to the number of observations (MSE, NLL) or the number of matched pairs (C-Index)

**Table 7:** Performance of models trained on the full data set and evaluated on the test set

Measure	Value	Sample size^a^	Response	Model
C-Index	0.554	3354	CDP	predictive
C-Index	0.608	5606	relapse	predictive
Average NLL	0.423	314	CDP	predictive
Average NLL	0.821	314	relapse	predictive
MSE	0.131	314	CDP	predictive
MSE	0.784	314	relapse	predictive

*CDP* confirmed disability progression, *C-Index* Harrell’s concordance statistic, *MSE* mean squared error, *NLL* negative log-likelihood.

^a^ Refers either to the number of observations (MSE, average NLL) or the number of matched pairs (C-Index)

**Table 8:** Performance of the predictive models based on leave-one-site-out cross-validation

Measure	Out-of-sample^a^ mean	In-sample^b^ mean	Sample size^c^	Response	Model
C-Index	0.579	0.652	307,784	CDP	predictive
C-Index	0.646	0.675	505,724	Relapse	predictive
MSE	0.125	0.119	3119	CDP	predictive
MSE	0.748	0.711	3119	Relapse	predictive
NLL	1254.330	1192.544	3119	CDP	predictive
NLL	2581.261	2523.731	3119	Relapse	predictive

*CDP* confirmed disability progression, *C-Index* Harrell’s concordance statistic, *MSE* mean squared error, *NLL* negative log-likelihood.

^a^ Based on predictions for patients from a clinical site not used during training

^b^ Based on predictions for all patients from all clinical sites used for training

^c^ Refers either to the number of observations (MSE, NLL) or the number of matched pairs (C-Index)

##### 10-fold cross-validation and test set

For 10-fold cross-validation, the percentage change between in-sample and out-of-sample performance measures is always less than 9.5%. For most performance measures and models, this change is even less than 5%. The percentage change between out-of-sample and test-set performance measures is less pronounced. For the relapse model, the MSE and the negative log-likelihood drop less than 2% and the C-Index drops by 5.7%. For the CDP model, the three performance measures drop by 5.7–6.3%.

As the models presented in this study should be applicable to different time windows, performance measures were additionally evaluated for forecast windows between 0.5 and 5.5 years. All performance measures show the best results in the first half year, remain stable in the time window between 0.5 and 4.5 years and decline for time windows above 4.5 years. The time windows (0.5, 1.5], (1.5, 2.5], (2.5, 3.5], (3.5, 4.5] show comparable results for all performance measures (Additional file 4: Table S4.1).

##### Leave-one-site-out cross-validation

Only a slight increase in negative log-likelihood is observed when making predictions for new clinical sites compared with in-sample predictions. Furthermore, the negative log-likelihood has a similar variability within and across clinical sites for both the relapse and CDP models (Additional file 9). These findings indicate that the models generalize well to new clinical sites.

In summary, the results presented in this section indicate that the relapse model is more robust than the CDP model and that both predictive models are robust and therefore able to generalize to new patients and clinical sites, especially when predicting up to 4.5 years into the future.

#### Comparison with nested models

Two additional nested models of lower complexity were presented in Section 2.3.4. Predictive models were compared with these non-personalized and prognostic models based on out-of-sample performance measures (40 times 10-fold cross-validation).

##### Comparison between predictive and non-personalized model

When adding personal characteristics to the relapse model, an increase of performance is observed in both discrimination (increase of C-Index from 0.5531 to 0.6458 by 0.0927) and goodness-of-fit (decrease of negative log-likelihood of − 69.3), while no change is observed for the MSE (− 0.0003). For the CDP model, the results derived from the performance measures are inconclusive. The negative log-likelihood and the MSE show a decrease in performance from the non-personalized to the predictive model (5.8 resp. 0.00048 difference). On the other hand, the addition of the personal characteristics causes an improvement of the C-Index by 0.0352.

##### Comparison between predictive and prognostic model

The predictive models include additional parameters accounting for interactions between patient characteristics and index therapy compared with the prognostic model (Table 3**),** which allows the predicted therapy effect to differ for different patients (Section 2.3.4). For the relapse model, a decrease in performance is observed when accounting for interactions (C-Index − 0.0024, MSE 0.0242, negative log-likelihood 6.2). For the CDP model, a decrease is observed in goodness-of-fit (MSE 0.00011, negative log-likelihood − 1.4) while an increase in performance is observed for the C-Index (0.0194).

In summary, adding personal characteristics as predictors is beneficial in terms of C-Index and negative log-likelihood, for the relapse model, and in terms of C-Index, for the CDP model. These findings suggest that this first level of personalization is worth to be further investigated. Adding a second level of personalization, i.e. interactions between patient characteristics and index therapy, increases model complexity and shows no improvement in the performance measures for the relapse model, and an improvement in the C-Index and negative log-likelihood of the CDP model. In order to further assess the benefit of the predictive models, a new method was introduced in Section 2.3.6 and is evaluated below in Section 3.3.5. This allows to investigate the potential of these models from a different perspective.

#### Model robustness

It was shown that the predictive models are robust against different choices of the priors, as for the vast majority of patients the changes in the model coefficients did not affect the therapy ranking (Additional file 5: Figure S5.7). Exceptions were cases where the predictions for different therapies were already similar. Similarly, the predictive models proved robust against differing characteristics of the underlying patient population and against changes in the sample size within the assessed range (Additional file 6 and Additional file 7).

#### Comparison of predicted therapy response

Both predictive models enable to derive a personalized ranking of therapies for each patient (Section 2.3.6). In the following, disease activity is compared within a survey-weighted GLM between patients being treated with the highest ranked therapy DMT* and patients treated with a different DMT.

From the six therapies discussed in this study (Dimethylfumarat, Fingolimod, Glatirameracetat, Interferon-ß1, Natalizumab, and Teriflunomide) only those which had both patients receiving and not receiving DMT* could be considered. Furthermore, implementation properties of the employed packages required a minimum of 10 observations per condition [20]. Due to these criteria, Glatirameracetat, Interferon-ß1 and Teriflunomide had to be excluded from the assessment of the relapse model and Interferon-ß1 had to be excluded from the assessment of the CDP model (Additional file 8: Table S8.1).

The results of the relapse model are presented in Table 9. This table summarizes the group comparisons derived from the survey-weighted negative binomial GLM svyglm.nb (Section 2.3.6), applied to the observed number of relapses and for the respective DMT*. Observation time has been accounted for within this GLM. As the indicator variable takes value 1 for receivers of DMT* and 0 for non-receivers of DMT*, a negative slope indicates a lower disease activity for patients who received DMT*. Both Dimethylfumarat and Natalizumab are associated with a significantly lower number of observed relapses at the *p* < 0.05 significance level when patients received DMT* compared to other DMTs. Fingolimod, however, is associated with lower sample sizes in both groups, and the differences between the two groups are not significant.

**Table 9:** Comparison of therapy effectiveness for the relapse model

DMT*	Slope coefficient^a^	Sample size when DMT* was taken	Sample size when DMT* was not taken	*p*-value
Dimethylfumarat	**−0.6918**	**134**	**307**	**0.016**
Fingolimod	0.0423	128	112	0.860
Natalizumab	**−0.4376**	**234**	**2182**	**0.019**

DMT* highest ranked disease modifying therapy.

^a^ Derived from a survey-weighted negative binomial generalized linear model where negative sign indicates lower disease activity

The results of the CDP model are presented in Table 10. This table summarizes the group comparisons derived from the survey-weighted quasi-binomial GLM svyglm (Section 2.3.6), applied to the observed occurrences of CDP, and for the respective DMT*. Analogously to the assessment of the relapse model, observation time has been accounted for within this GLM and a negative slope indicates a lower disease activity for patients who received DMT*. All therapies except Fingolimod are associated with a lower occurrence of CDP when patients received DMT*, although only the differences for Dimethylfumarat are statistically significant at the significance level of *p* < 0.05. The remaining therapies are associated with considerably smaller sample sizes in at least one of the two groups.

**Table 10:** Comparison of therapy effectiveness for the CDP model

DMT*	Slope coefficient^a^	Sample size when DMT* was taken	Sample size when DMT* was not taken	*p*-Value
Dimethylfumarat	**−0.5363**	**863**	**306**	**0.027**
Fingolimod	0.1114	135	87	0.792
Glatirameracetat	−0.5336	238	45	0.350
Natalizumab	−0.4021	1101	132	0.405
Teriflunomide	−0.4317	179	16	0.730

DMT* highest ranked disease modifying therapy.

^a^ Derived from a survey-weighted quasi-binomial generalized linear model where negative sign indicates lower disease activity

In summary, recommendations based on predicted rankings could not be assessed for all six therapies due to sample size (Additional file 8: Table S8.1). For the CDP model, five of the six therapies could be assessed, where a negative estimated slope from a survey-weighted GLM corresponds to the highest ranked therapy in four cases, and is significant for one comparison. For the relapse model, three of the six therapies were assessed, where a negative estimated slope from a survey-weighted GLM is associated with the highest ranked therapy in all cases except for Fingolimod, and is significant for two comparisons. These results indicate that although performance measures do not capture the additional benefit of interactions between patient characteristics and index therapy, the comparison of treatment effectiveness derived from both predictive models adds value in clinical practice, as patients receiving DMT* show less disease activity than patients receiving any other DMT (significant for three out of eight comparisons).

## Discussion

A framework for personalized prediction was employed to assess the effectiveness of different DMTs with regards to clinical outcomes of RRMS. Hierarchical Bayesian GLMs were implemented to predict the number of relapses or the occurrence of a CDP, for several available DMTs. The predictive framework was based on real-world data collected in the NTD registry, which consists of clinical data on patient characteristics and disease history.

Assessment of the model performance using established statistical methods demonstrated that the relapse model and the CDP model provide robust and accurate predictions, and that both models generalize to new patients and clinical sites.

The predictive relapse model achieved an average out-of-sample C-Index of 0.65 and an average out-of-sample MSE of 0.76 relapses. The predictive CDP model achieved an average C-Index of 0.58 and an average out-of-sample MSE of 0.12 CDPs.

The predictive models were shown to be robust against different choices of the priors and against sample size. Robustness against different choices of the priors was proven by the fact that changing the prior distributions does not influence the predicted therapy ranking. Exceptions were cases where the predictions for different therapies were already similar. Robustness against sample size was proven by the fact that model coefficients do not change significantly when reducing the sample size within the assessed range.

The overall performance of the predictive models allows for reliable comparisons of the effectiveness of different therapies. Therapy rankings obtained from both predictive models proved to be aligned with real-world clinical outcomes. Patients having received the therapy with the highest predicted effectiveness were less likely to suffer from relapses or to experience a CDP compared with patients treated with other DMTs. Statistically significant differences were always associated with better disease outcomes for patients who received the highest ranked therapy.

The generalizability of the models was evaluated by implementing a 10-fold cross-validation scheme, a leave-one-site-out cross-validation scheme with respect to the clinical site, and by using a test set. When comparing in-sample against out-of-sample performance measures, performance never dropped by more than 9.5% for all three performance measures used in this study, indicating that the predictive models generalize well to an unseen patient population. Comparable performance was obtained using 10-fold cross-validation and the test set. This indicates that the predictive models can be applied to new patients that fulfill the inclusion criteria. The availability of a large multi-site dataset with high-quality entries in the NTD registry proved important for testing the generalizability of the models to new clinical sites, an inevitable requirement to ensure applicability to a wide range of patients.

As application of predictive models in RRMS is still at an early stage, this study builds on previous studies in this field ([8, 9, 21, 22]) and addresses identified challenges: (1) effectiveness of different therapies with regards to disease course can be compared with one another as they are based on the same model and underlying data, (2) the models fulfill multiple established statistical criteria for accuracy and robustness, and show comparable performance for predictions up to 4.5 years into the future, (3) the models generalize to new patients and new clinical sites, (4) model predictions are clinically meaningful, since patients receiving the therapy with the highest predicted effectiveness had a better observed clinical outcome in terms of number of relapses and CDP.

The Bayesian predictive modeling setup is ideal for including newly available therapies, as results derived from clinical studies can be used as prior information. Predictive models will be updated on a regular basis every three months, following the continuous updates of the NTD registry. For this, the predictive models are fitted based on an updated training set after quality and inclusion criteria have been applied to a new data extraction, and the main validation routine as described above is performed. After quality checks have been passed, coefficients are updated accordingly. This will ensure that the underlying algorithms are monitored and validated regularly, and that changes in the RRMS treatment landscape will be captured as soon as possible. In addition, new models will be assessed to improve the capabilities of the framework even further.

For the presented and all further extensions of the predictive models, additional validation on external data sets is planned. As magnetic resonance imaging (MRI) and molecular data for the prediction of RRMS treatment response become available, their usefulness will be evaluated to improve predictive accuracy and tailor the predictive framework even further to personalized information.

## Conclusion

The findings presented in this study support a personalized approach in RRMS treatment and tackle some caveats in classical medicine, where assessing the potential individual benefit of therapy choices is based on group-level analysis and not on individual patient characteristics [23] [24] [25] [26]. Predictive models that are continuously updated and that provide personalized comparative therapy effectiveness insight support the multifaceted shared decision process between doctors and their patients in a clinically meaningful way. Positive experience of their use in daily clinical practice will foster collection of high-quality real-world data and contribute to the transition to effective personalized treatment of RRMS.

## Supplementary information

**Additional file 1.** “Inclusion criteria and resulting patient population”: Supplementary tables and results.

**Additional file 2.** “CDP definition”: Definition of confirmed disability progression.

**Additional file 3.** “Mathematical model description”: Supplementary information to Section 2.2.

**Additional file 4.** “Performance by observation time window”: Supplementary table.

**Additional file 5.** “Model robustness against different prior choices”: Supplementary methods and results.

**Additional file 6.** “Model robustness against differences in patient characteristics”: Supplementary methods and results.

**Additional file 7.** “Model robustness against different training set size”: Supplementary methods and results.

**Additional file 8.** “Comparison of predicted treatment effectiveness”: Supplementary tables.

**Additional file 9.** “Clinical site effect”: Supplementary figures.

## Data Availability

The datasets generated and analyzed during the current study are not publicly available due to them containing information that could compromise research participant privacy but are available from the corresponding author SB on reasonable request.

## Abbreviations

CDP: Confirmed disability progression
DMF: Dimethylfumarat
DMT: Disease-modifying therapy
EDSS: Expanded Disability Status Scale
FTY: Fingolimod
GA: Glatirameracetat
GLM: Generalized linear model
IBE: Institut für medizinische Informationsverarbeitung, Biometrie und Epidemiologie
IF: Interferon-ß1
MAD: Median absolute deviation
MRI: Magnetic resonance imaging
MS: Multiple sclerosis
MSE: Mean squared error
NA: Natalizumab
NLL: Negative log-likelihood
NTD: NeuroTransData
RCT: Randomized clinical trial
RRMS: Relapsing-remitting multiple sclerosis
SE: Standard error
TERI: Teriflunomide
