# Supplementary material for: Framework for personalized prediction of treatment response in relapsing remitting multiple sclerosis
Source: BMC Med Res Methodol. 2020 Feb 7;20:24. doi: 10.1186/s12874-020-0906-6 (PMC7006411; doi:10.1186/s12874-020-0906-6)
Supplement: Supplementary file 1 — Additional file 1. “Inclusion criteria and resulting patient population”: Supplementary tables and results. [file 12874_2020_906_MOESM1_ESM.pdf]

Additional file 1: Inclusion criteria and resulting patient population

Descriptive statistics about the patient population can be found in **Table S1.2** and **Table S1.3**. Both tables look at patient data at two stages of the data filtering process. More specifically, they provide numbers about the data (i) before the last 14 filtering criteria have been applied, and (ii) after it has been fully filtered. For a list of all filtering criteria see **Table S1.1**.

First, consider the patient data that results from the application of all filters, i.e. the data that our model estimates are based on. Among the most interesting statistics that describe it, note that the median number of relapses on index therapy is 0, which means that a majority of patients do not suffer a relapse in this therapy cycle. Also, in 16% of the cases a CDP is observed after the start of the index therapy. For what regards the predictors' distribution, one observes that two thirds of the patient population is aged between 30 and 50, that almost half of the patients have an EDSS lower than 1.5, and that 75% of them are females. Also, note that among all possible index therapy types we retained only six, namely Dimethylfumarat (22%), Fingolimod (25%), Glatirameracetat (13%), IF-beta1 (19%), Natalizumab (8%), and Teriflunomide (13%). This way, predictions can be based on at least 266 therapy cycles per DMT. Further details can be found in **Table S1.2** and **Table S1.3**.

Figure S1.1 displays boxplots for the time (in years) that elapsed between the MS diagnosis and the start of the index therapy, both marginally and given a certain variable (e.g. per age category, per therapy type, etc.). **Figure S1.2** displays the same kind of boxplots for the duration (in years) of the current therapy.

As can be seen in **Table S1.3**, application of the inclusion criteria in the filtering process (**Table S1.1**) leaves the characteristics of the underlying patient population globally unchanged. There are, however, a few exceptions to this. The graphs in **Figure S1.3** illustrate some of these changes.

Table S1.4 provides an overview over the number of patients per clinical site after application of the inclusion criteria. Overall our dataset contains patient data from 67 clinical sites, ranging from large (n=247) to very small sites (n=3).

Table S1.1: Quality and inclusion criteria overview

Description	ID	Therapies	
		Loss	Total
Original entries	1		102337
Removing duplicates	2	-1758	100579
Removing cycles used in clinical studies	3	-1566	99013
Missing diagnosis Date	4	-391	98622
Missing date of Birth	5	0	98622
Recorded date (e.g. birth date) after data delivery date	6	-53	98569
Date Of birth before 1916	7	0	98569
Recorded date (e.g. start of therapy cycle) before date of birth	8	-803	97766
Remove patients that have a therapy with missing start Date	9	-79	97687
Remove patients with therapy end date before start date	10	-462	97225
Remove therapies corresponding to patients with no EDSS visits	11	-7900	89325

Remove therapies with start date after last visit	12	-2218	87107
Therapies that were stopped within a day (duration = 0 days)	13	-153	86954
Insert therapy cycle “NoDMT” to patients where no therapies are recorded	14	989	87943
Merge therapies with the same start date	15	-8080	79863
Add missing end dates	16	-193	79670
Remove total overlap with NoDMT	17	-25256	54414
Merge overlapping equal therapies	18	-587	53827
Merge overlapping different therapies (therapy name changes to “Other DMT”)	19	-1921	51906
Add NoDMT cycles into gaps where no therapy was recorded	20	20963	72869
Remove NoDMT cycles after DMT with duration less than 92 days (wash out phase)	21	-5823	67046
Merge subsequent therapies and fill therapy gaps of duration less than 92 days	22	-24346	42700
Locking away test set[1)]	23	-2140	40560
Filter: Not RRMS	24	-6446	34114
Filter: Therapy cycle started before 2009-01-01	25	-13386	20728
Filter: Therapy cycle started when patient was under 18	26	-278	20450
Filter: Index therapy is NoDMT	27	-7988	12462
Filter: EDSS at start of therapy cycle > 6	28	-124	12338
Filter: Observed ARR is > 12	29	-26	12312
Filter: First therapy cycle	30	-321	11991
Filter: Therapy cycle that started within 6 months after diagnosis	31	-2578	9413
Filter: Index therapy is OtherDMT	32	-24	9389
Filter: Missing EDSS measurement	33	-3754	5635
Filter: Response could not be computed	34	0	5635
Filter: Cycles without previous relapse	35	-1281	4354
Filter: Too few observations (visits) for this therapy	36	-327	4027
Filter: Current Therapy is OtherDMT	37	-47	3980
Filter: More than one therapy cycle per patient (randomly sample one cycle)	38	-857	3123
Filter: Clinical sites where only one patient is left	39	-4	3119

Overview of quality and inclusion criteria, where the steps in the first half of the table represent data cleaning (quality), and in the second half represent filtering (inclusion).

[1)] Note that work on this project started with a database extraction in March 2016. At that time the initial test set (10% of all therapy cycles) was randomly selected after applying all filters. Since 2017 the approach has been different: 10% of all newly available therapy cycles with each updated database extraction are now randomly sampled before applying the inclusion criteria. As the previous sampling of the test set remains unchanged, the loss at this step therefore appears to be less than 10%. However, after the remaining filters are applied to the test set, it contains 314 therapy cycles, which approximately represents 10% of the training set size (n = 3119).

Table S1.2: Responses overview

Response	Summary	Before filtering (n=20728)	After filtering (n=3119)
Number of relapses (count)	Min	0	0
	1st quartile	0	0
	Median	0	0
	Mean	0.54	0.43
	3rd quartile	1	1
	Max	14	7
	Standard deviation	0.95	0.88
CDP (binary)	Mean	0.11	0.16

Summarized responses’ distributions before and after application of the inclusion criteria.

Table S1.3: Predictors overview

Predictor	Summary	Before filtering (n=20728)	After filtering (n=3119)
Duration of index therapy (yrs)	Min	0.00	0.08
	1st quartile	0.36	0.77
	Median	1.19	1.92
	Mean	1.90	2.38
	3rd quartile	2.88	3.60
	Max	9.43	9.39
	Standard deviation	1.96	1.95
Duration of current therapy (yrs)	Min	0.00	0.02
	1st quartile	0.02	0.84
	Median	0.61	2.07
	Mean	2.23	3.85
	3rd quartile	2.58	5.04
	Max	38.41	35.36
	Standard deviation	3.91	4.65
Relapses count	Min	0	0
	1st quartile	0	0
	Median	0	0
	Mean	0.56	0.64
	3rd quartile	1	1
	Max	6	6
	Standard deviation	0.76	0.76
Diagnosis distance (yrs)	Min	0.00	0.10
	1st quartile	0.02	2.81
	Median	2.24	6.38
	Mean	5.05	8.14
	3rd quartile	8.30	11.84
	Max	52.60	47.18
	Standard deviation	6.47	6.69
Age	(0,18]	1%	-
	(18,30]	27%	19%
	(30,40]	31%	31%
	(40,50]	27%	33%
	50+	14%	17%
EDSS	(0,1.5]	49%	47%
	(1.5,2.5]	23%	24%
	(2.5,3.5]	12%	14%
	(3.5,10]	16%	15%
Current therapy	NoDMT	51%	51%
	Alemtuzumab	0%	-
	Azathioprin	0%	-
	Cladribin	0%	-
	Cyclophosphamid	0%	-
	Daclizumab	1%	-
	Dimethylfumarat	4%	3%
	Fingolimod	4%	2%

	Glatirameracetat	10%	12%
	IF-beta1	22%	26%
	Immunglobine	0%	-
	Laquinimod	0%	-
	Methotrexat	0%	-
	Mitoxantron	0%	-
	Natalizumab	4%	4%
	Ocrelizumab	0%	-
	OtherDMT	0%	-
	Rituximab	0%	-
	Siponimod	0%	-
	Teriflunomide	2%	2%
Index therapy	NoDMT	39%	-
	Alemtuzumab	1%	-
	Azathioprin	0%	-
	Cladribin	0%	-
	Cyclophosphamid	0%	-
	Daclizumab	1%	-
	Dimethylfumarat	9%	22%
	Fingolimod	9%	25%
	Glatirameracetat	10%	13%
	IF-beta1	20%	19%
	Immunglobine	0%	-
	Laquinimod	0%	-
	Methotrexat	0%	-
	Mitoxantron	0%	-
	Natalizumab	4%	9%
	Ocrelizumab	0%	-
	OtherDMT	0%	-
	Rituximab	0%	-
	Siponimod	0%	-
	Teriflunomide	5%	13%
Gender	F	74%	75%
	M	26%	25%
Second-line therapy indicator	FALSE	90%	88%
	TRUE	10%	12%
DMTs count	0	51%	23%
	1	27%	49%
	2	13%	18%
	3+	9%	10%
Relapse distance	[0, 0.25)	47%	17%
	[0.25,1)	17%	32%
	[1, 3)	15%	17%
	[3, Inf)	21%	24%

Summarized predictors’ distributions before and after application of the inclusion criteria. Note that the duration of the index therapy is included as an offset into the models to account for varying therapy cycle durations.

Table S1.4: Clinical sites overview

ID	Frequency	ID	Frequency	ID	Frequency
Site ID 1	247	Site ID 24	54	Site ID 47	17
Site ID 2	211	Site ID 25	51	Site ID 48	16
Site ID 3	143	Site ID 26	50	Site ID 49	14
Site ID 4	137	Site ID 27	49	Site ID 50	12
Site ID 5	131	Site ID 28	46	Site ID 51	11
Site ID 6	121	Site ID 29	45	Site ID 52	11
Site ID 7	94	Site ID 30	43	Site ID 53	11
Site ID 8	90	Site ID 31	42	Site ID 54	10
Site ID 9	83	Site ID 32	40	Site ID 55	10
Site ID 10	81	Site ID 33	38	Site ID 56	10
Site ID 11	80	Site ID 34	35	Site ID 57	9
Site ID 12	77	Site ID 35	34	Site ID 58	6
Site ID 13	71	Site ID 36	31	Site ID 59	5
Site ID 14	70	Site ID 37	28	Site ID 60	5
Site ID 15	69	Site ID 38	24	Site ID 61	5
Site ID 16	65	Site ID 39	22	Site ID 62	5
Site ID 17	62	Site ID 40	22	Site ID 63	4
Site ID 18	61	Site ID 41	21	Site ID 64	4
Site ID 19	60	Site ID 42	20	Site ID 65	3
Site ID 20	57	Site ID 43	19	Site ID 66	3
Site ID 21	57	Site ID 44	19	Site ID 67	3
Site ID 22	54	Site ID 45	19		
Site ID 23	54	Site ID 46	18		

Frequency of patients in clinical sites after application of the inclusion criteria.

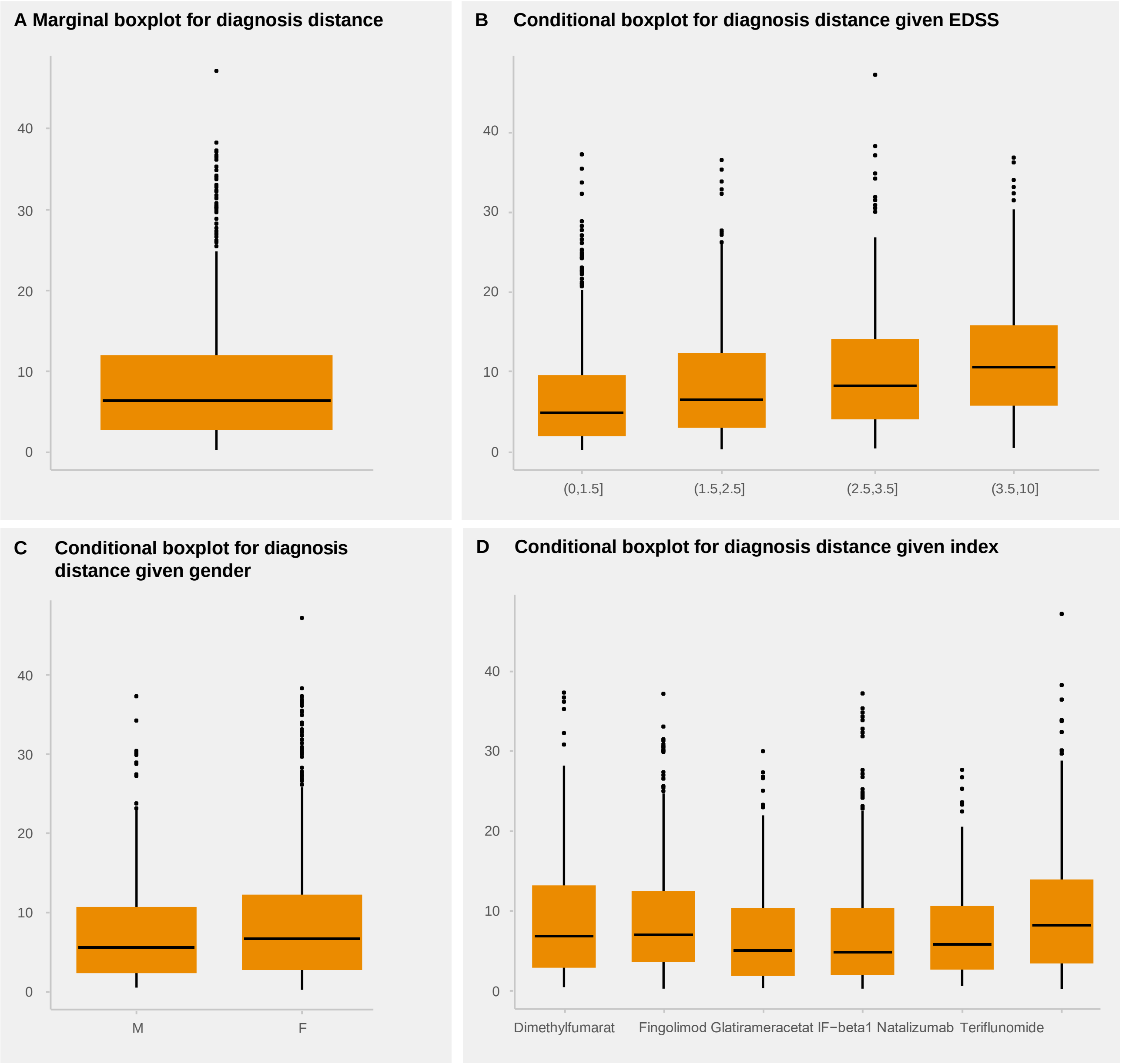

Figure S1.1: Boxplot summarizing the distribution of the time (in years) that elapsed between the date of the MS diagnosis and the start of the index therapy, marginally (A) or given the EDSS score (B), the gender (C) or the index therapy type (D).

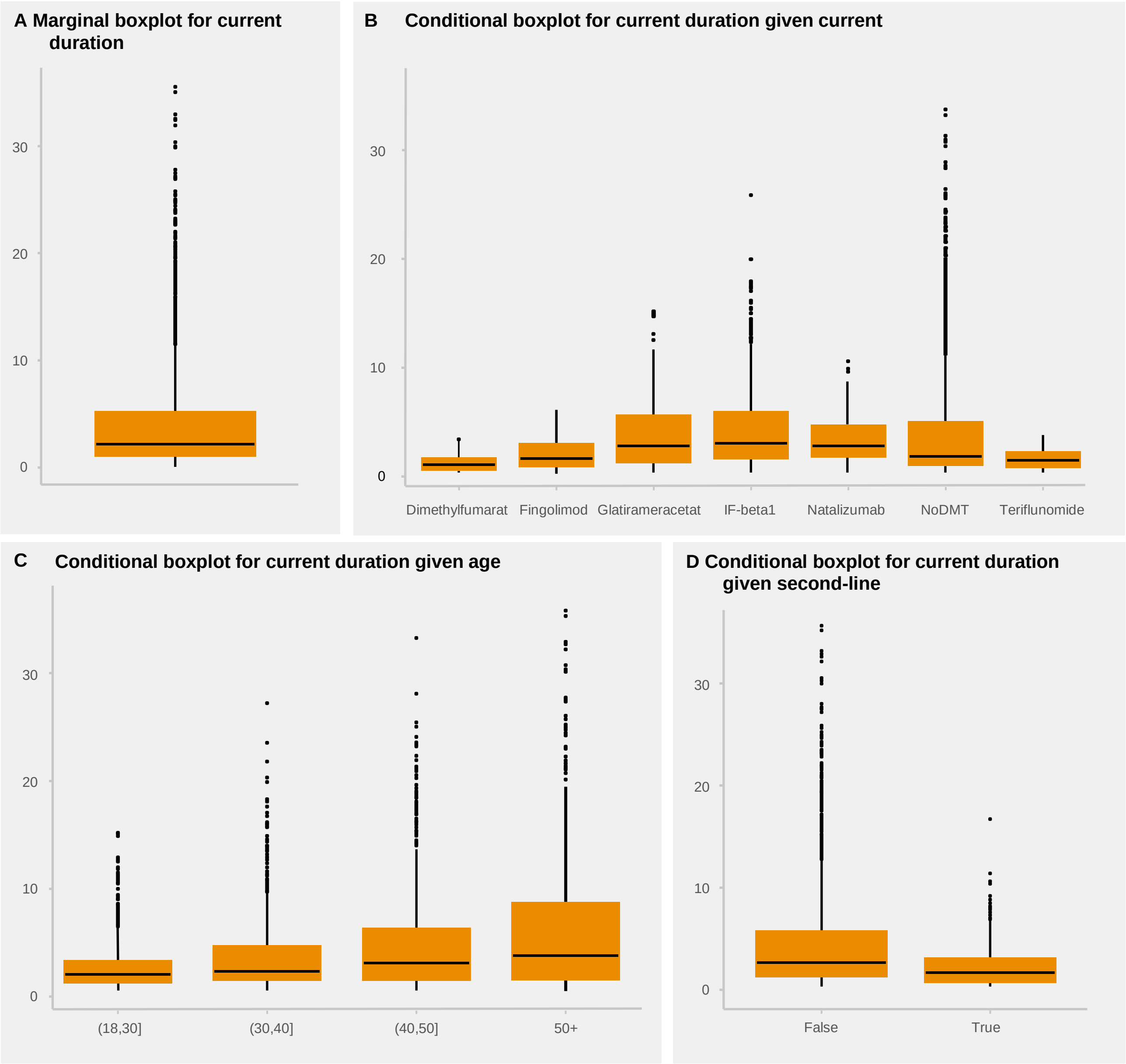

Figure S1.2: Boxplot summarizing the distribution of the duration (in years) of the current therapy, marginally (A) or given the current therapy type (B), the age (C) or the indicator as to whether a second-line DMT has been taken in the past (D).

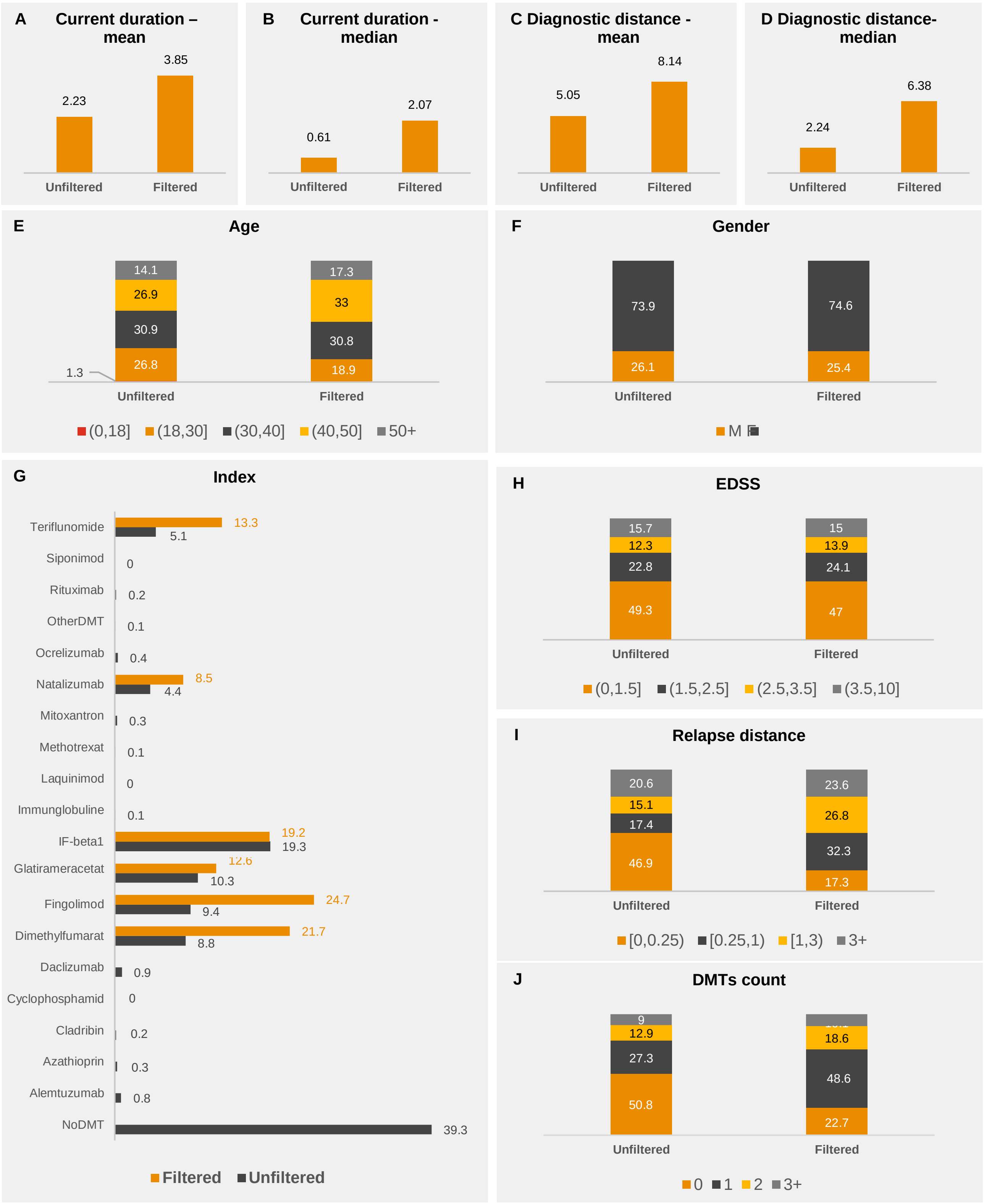

Figure S1.3: Behavior of a selection of predictors under filtering. Are inspected: the duration of the current therapy (A-B), the diagnosis distance (C-D), the patient's age (E), the patient's gender (F), the index therapy type (G), the EDSS (H), the relapse distance (I), and the DMTs count (J). In particular, after application of the inclusion criteria, the following changes were observed: the mean duration of current therapy increases (A); the average diagnosis distance increases (C); most often shorter than three months before filtering, the relapse distance most often lies between three months and one year after filtering (I); the number of patients having undergone exactly one DMT becomes a majority (J). No additional significant changes were observed.
