# Supplementary material for: Framework for personalized prediction of treatment response in relapsing remitting multiple sclerosis
Source: BMC Med Res Methodol. 2020 Feb 7;20:24. doi: 10.1186/s12874-020-0906-6 (PMC7006411; doi:10.1186/s12874-020-0906-6)
Supplement: Supplementary file 2 — Additional file 2. “CDP definition”: Definition of confirmed disability progression. [file 12874_2020_906_MOESM2_ESM.pdf]

Additional file 2: CDP definition

A CDP is defined by an increase in EDSS during a therapy cycle, where all the following criteria are met:

a) there is an increase in EDSS in comparison with the previous EDSS measurement (this is either the therapy baseline EDSS or an on-therapy measurement), and the increase passes a pre-defined threshold,

b) the increase is maintained for at least 12 weeks (that is, any other EDSS measurement within 12 weeks also passes the pre-defined threshold),

c) the increase is confirmed by at least one EDSS measurement i) 12 weeks after this increase and no later than one year after the therapy cycle, and ii) this measurement is not associated with a relapse.

The pre-defined threshold depends on the previous EDSS measurement: if the EDSS is ≤ 5.5, the increase must be at least 1.0 point; otherwise the increase must be at least 0.5 points. If all of the criteria are fulfilled, the time of the CDP is the date of the visit that the progression is associated with.
