# Supplementary material for: Framework for personalized prediction of treatment response in relapsing remitting multiple sclerosis
Source: BMC Med Res Methodol. 2020 Feb 7;20:24. doi: 10.1186/s12874-020-0906-6 (PMC7006411; doi:10.1186/s12874-020-0906-6)
Supplement: Supplementary file 3 — Additional file 3. “Mathematical model description”: Supplementary information to Section 2.2. [file 12874_2020_906_MOESM3_ESM.pdf]

Additional file 3: Mathematical model description

The density of a random variable following a negative binomial distribution with mean μ and shape ϕ is given by

$$f(y) = \binom{y+\phi-1}{y}\left(\frac{\mu}{\mu+\phi}\right)^{y}\left(1-\frac{\mu}{\mu+\phi}\right)^{\phi}$$

with $y \in \{0, 1, 2, \dots\}$. In the relapse model, the number of on-therapy relapses is assumed to follow such distribution. The density of a random variable following a Bernoulli distribution with mean μ is given by

$$f(y) = \mu^{y}\,(1-\mu)^{1-y}$$

with $y \in \{0, 1\}$. In the CDP model, the occurrence of an EDSS progression (which takes value 1 if a progression is observed over the course of the index therapy, and 0 otherwise) is assumed to follow such a distribution.

Both models are Bayesian generalized linear mixed models, that is, the following specifications are made:

1) In order to capture within-clinic correlation, clinic-specific random intercepts $b_i \sim N(0, \sigma^2), i = 1, \dots, m$ are introduced, where m denotes the number of clinical sites. We then write $\boldsymbol{b} := (b_1, \dots, b_m)$.
2) The response variables $y_k, k = 1, \dots, N$, are assumed to be independent conditionally on $\boldsymbol{b}$, and their link-transformed conditional expectation is assumed to be given by $g\big(E(y_k \mid \beta, \boldsymbol{b})\big) = \beta' x_k + b_{i(k)} + \log(t_k)$ where x_k denotes the set of characteristics of the k-th patient, $i(k)$ his/her clinic assignment, and t_k the duration of his/her index therapy. The link is chosen to be $g = \log$ in the relapse model, while it is defined as $g = \text{logit}$ in the CDP model.
3) Specific prior distributions are assigned to the random parameters β and σ^2, and additionally to ϕ in the relapse model.

In both the relapse and the CDP model, the choice of log-offset described in (2) is motivated by the following properties. In the relapse model, it results in an expected number of relapses (given the fixed and the random effects) that is linear with respect to the duration of the index therapy. Indeed, one has

$$E(y_k \mid \beta, \boldsymbol{b}) = t_k \cdot e^{\beta' x_k + b_{i(k)}}$$

In the CDP model, the choice of a log-offset yields the following probability of an EDSS progression:

$$\Pr(y_k = 1 \mid \beta, \boldsymbol{b}) = E(y_k \mid \beta, \boldsymbol{b}) = \frac{t_k \cdot e^{\beta' x_k + b_{i(k)}}}{1 + t_k \cdot e^{\beta' x_k + b_{i(k)}}}$$

Looking at it as a function of t, one observes that the map satisfies two desirable boundary conditions, that is, the probability of CDP is zero at the very beginning of the therapy ($t = 0$), and it is almost 1 for long therapy durations. Moreover, for small likelihood values, it behaves approximately like a linear function of the therapy duration.
