# Supplementary material for: Framework for personalized prediction of treatment response in relapsing remitting multiple sclerosis
Source: BMC Med Res Methodol. 2020 Feb 7;20:24. doi: 10.1186/s12874-020-0906-6 (PMC7006411; doi:10.1186/s12874-020-0906-6)
Supplement: Supplementary file 4 — Additional file 4. “Performance by observation time window”: Supplementary table. [file 12874_2020_906_MOESM4_ESM.pdf]

Additional file 4: Performance by observation time window

Table S4.1: Performance by observation time window.

Sample	n	Value	Measure	Response	Observation time (yrs)
out-of-sample	18037	0.725730443	C-Index	CDP	(0,0.5]
out-of-sample	98518	0.603605433	C-Index	CDP	(0.5,1.5]
out-of-sample	67977	0.591266164	C-Index	CDP	(1.5,2.5]
out-of-sample	62006	0.526626456	C-Index	CDP	(2.5,3.5]
out-of-sample	42715	0.566885169	C-Index	CDP	(3.5,4.5]
out-of-sample	9527	0.585913719	C-Index	CDP	(4.5,5.5]
out-of-sample	9004	0.58251888	C-Index	CDP	(5.5,Inf]
out-of-sample	58563	0.745009648	C-Index	Relapse	(0,0.5]
out-of-sample	185104	0.630486105	C-Index	Relapse	(0.5,1.5]
out-of-sample	102662	0.656304183	C-Index	Relapse	(1.5,2.5]
out-of-sample	85546	0.629129357	C-Index	Relapse	(2.5,3.5]
out-of-sample	53584	0.621668782	C-Index	Relapse	(3.5,4.5]
out-of-sample	10990	0.555277525	C-Index	Relapse	(4.5,5.5]
out-of-sample	9275	0.623234501	C-Index	Relapse	(5.5,Inf]
out-of-sample	555	0.022519098	MSE	CDP	(0,0.5]
out-of-sample	769	0.089770155	MSE	CDP	(0.5,1.5]
out-of-sample	532	0.126785811	MSE	CDP	(1.5,2.5]
out-of-sample	431	0.170392868	MSE	CDP	(2.5,3.5]
out-of-sample	406	0.176183778	MSE	CDP	(3.5,4.5]
out-of-sample	167	0.203516824	MSE	CDP	(4.5,5.5]
out-of-sample	259	0.234945142	MSE	CDP	(5.5,Inf]
out-of-sample	555	0.096080014	MSE	Relapse	(0,0.5]
out-of-sample	769	0.462789086	MSE	Relapse	(0.5,1.5]
out-of-sample	532	0.751013541	MSE	Relapse	(1.5,2.5]
out-of-sample	431	0.856222137	MSE	Relapse	(2.5,3.5]
out-of-sample	406	0.986274177	MSE	Relapse	(3.5,4.5]
out-of-sample	167	1.547045736	MSE	Relapse	(4.5,5.5]
out-of-sample	259	1.988639802	MSE	Relapse	(5.5,Inf]
out-of-sample	555	56.86443123	Negative log-likelihood	CDP	(0,0.5]
out-of-sample	769	251.7976131	Negative log-likelihood	CDP	(0.5,1.5]
out-of-sample	532	220.8054915	Negative log-likelihood	CDP	(1.5,2.5]
out-of-sample	431	229.2835966	Negative log-likelihood	CDP	(2.5,3.5]
out-of-sample	406	216.6877741	Negative log-likelihood	CDP	(3.5,4.5]
out-of-sample	167	99.7876417	Negative log-likelihood	CDP	(4.5,5.5]
out-of-sample	259	171.8032924	Negative log-likelihood	CDP	(5.5,Inf]
out-of-sample	555	173.0040932	Negative log-likelihood	Relapse	(0,0.5]
out-of-sample	769	580.7701446	Negative log-likelihood	Relapse	(0.5,1.5]

out-of-sample	532	465.7815193	Negative log-likelihood	Relapse	(1.5,2.5]
out-of-sample	431	420.5025399	Negative log-likelihood	Relapse	(2.5,3.5]
out-of-sample	406	398.8625403	Negative log-likelihood	Relapse	(3.5,4.5]
out-of-sample	167	205.0234432	Negative log-likelihood	Relapse	(4.5,5.5]
out-of-sample	259	339.0155422	Negative log-likelihood	Relapse	(5.5,Inf]

The model performance measured by C-index, MSE and negative log-likelihood is shown for out-of-sample predictions of the predictive CDP and relapse models, and it is based on 10-fold cross-validation. The integer in the ‘n’ column refers either to number of observations (MSE, Negative log-likelihood) or the number of matched pairs in that time interval (C-Index). Therapy cycles are grouped according to their observation time, as can be seen in the ‘Observation time’ column.
