# Supplementary material for: Framework for personalized prediction of treatment response in relapsing remitting multiple sclerosis
Source: BMC Med Res Methodol. 2020 Feb 7;20:24. doi: 10.1186/s12874-020-0906-6 (PMC7006411; doi:10.1186/s12874-020-0906-6)
Supplement: Supplementary file 5 — Additional file 5. “Model robustness against different prior choices”: Supplementary methods and results. [file 12874_2020_906_MOESM5_ESM.pdf]

Additional file 5: Model robustness against different prior choices

1. Methods

All prior distributions were set to be weakly informative (Section 2.2). For the relapse model, four priors were taken into account, corresponding to the intercept, fixed effects, dispersion of the negative binomial distribution, and standard deviation of the random intercepts. For the CDP model, only three priors were needed, since there is no dispersion for this model.

In order to assess the robustness of the predictions with respect to a different choice of the priors, alternative posterior distributions were computed using other priors, and the resulting coefficients and predictions were compared to those obtained using the predictive models built with defaults priors (Section 2.2). A dependence of the prior-induced differences on the specific therapy under consideration was also investigated.

In particular, two different scenarios were considered. In the first, referred to as scenario A, wider prior distributions were used for the intercept, the fixed effects and, if present, the dispersion. In the second, referred to as scenario B, larger scale and shape parameters were used for the distribution of the standard deviation of the random intercepts, in addition to the changes described in scenario A. This allows to test the impact on the model of increasingly non-informative prior distributions and of different underlying Gamma distributions. A detailed description of the two scenarios and their comparison to the default priors is presented in **Table S5.1**.

2. Results

The impact of a different choice of the priors of the model is evaluated by comparing the resulting predictions with those obtained using the default priors. Additional studies on the impact of a different choice of the priors on predictions and model coefficients are reported below.

In order to check whether the choice of the priors induces a shift in the model predictions that is not uniform across therapies, and hence induces a change in the recommended therapy, the difference between the predictions of the default model and those of the alternative model are studied for each therapy separately. This is shown in **Figure S5.1**, which refers to scenario B of the relapse model (left) and CDP model (right), respectively. Analogous plots are obtained for the other scenarios. The median and the 25% and 75% quantiles for all scenarios are reported in **Table S5.2** for both relapse model and CDP model. The median of the prediction difference is positive for some therapies and negative for others, depending on the scenario, and is below 0.001 for all cases under consideration.

3. Additional studies

This section reports additional studies on the impact of a different choice of the priors on model coefficients and predictions.

Figure S5.2 shows the differences between the posterior medians of the fixed-effect parameters under the default priors and those medians under the priors of scenario A. **Figure S5.3** shows the posterior MADs of the fixed-effect parameters under the default priors and under the priors of scenario A. **Figure S5.4** shows the MADs of the random intercepts under the default priors and under the priors of scenario B.

The posterior medians of the parameters corresponding to the current therapy and to the interaction between the current therapy and its duration become larger in magnitude when using the alternative priors. The effect is more pronounced for the interaction terms. These parameters have the largest uncertainty, with MADs that even increase when moving from the default model to the alternative model. Thus, one might consider removing the corresponding predictors from the model, at least until more data are collected.

The histogram in **Figure S5.5** shows the difference between the predictions of the default model and those of the alternative model. The histogram in **Figure S5.6** shows a variation of the same histogram, where the percentage change with respect to the prediction of the default model is considered. The fact that the mean of the histograms is zero suggests that there is no bias induced by the prior distributions on the predictions. However, a percentage change of the order of 20-30% is observed from the plot in **Figure S5.6**.

For each model coefficient and each prior scenario, the credible interval corresponding to the 5% and 95% quantiles was computed. Two cross-checks were performed on these credible intervals. The first cross-check consisted in verifying that, if the credible interval obtained using the default priors contained (did not contain) zero, the same was true for the credible interval obtained using the alternative priors. The second cross-check consisted in verifying that the credible interval obtained using the alternative priors contained the median of the parameter's posterior distribution obtained using the default priors. Almost all model coefficients passed the two cross-checks. Exceptions were, for example, two parameters of the relapse model: the one associated with Teriflunomide as a current therapy, and the one corresponding to the interaction between the current therapy duration and Teriflunomide as a current therapy. The credible intervals of these two parameters contained zero under the default priors, and they did not contain zero under the priors of scenario B.

Table S5.1: Scenario overview for different prior choices: CDP and relapse model

Model	Scenario	Intercept	Fixed effects	Dispersion	Standard deviation of random intercepts
Relapse	Default priors	N(0, 10)	N(0, 2.5)	Half-Cauchy(0, 5)	Gamma(1, 1)
	Scenario A	N(0, 20)	N(0, 5)	Half-Cauchy(0, 10)	Gamma(1, 1)
	Scenario B	N(0, 20)	N(0, 5)	Half-Cauchy(0, 10)	Gamma(2, 2)
CDP	Default priors	N(0, 10)	N(0, 2.5)	-	Gamma(1, 1)
	Scenario A	N(0, 20)	N(0, 4)	-	Gamma(1, 1)
	Scenario B	N(0, 20)	N(0, 4)	-	Gamma(2, 2)

Table S5.2: Difference in predictions for the relapse and CDP model

Model	Scenario	Dimethylfumarat	Glatirameracetat	Natalizumab	Fingolimod	IF-beta1	Teriflunomide
Relapse	Scenario A	0.000667	0.000167	-0.000125	0.000375	0.000333	0.000750
		-0.00300	-0.00575	-0.00450	-0.00540	-0.00496	-0.00425
		0.00552	0.00500	0.00465	0.00640	0.00631	0.00579
Relapse	Scenario B	0.000750	0.000333	-0.0000417	-0.000250	0.000667	-0.000292
		-0.00308	-0.00508	-0.00508	-0.00523	-0.00390	-0.00483
		0.00592	0.00533	0.00417	0.00550	0.00665	0.00483
CDP	Scenario A	0.000750	-0.0000833	-0.000417	-0.000250	-0.000250	-0.000583
		-0.000417	-0.00142	-0.00275	-0.00183	-0.00150	-0.00233
		0.00225	0.00100	0.00125	0.00100	0.000833	0.000583
CDP	Scenario B	0.000917	0.000167	-0.000417	-0.0000417	-0.0000833	-0.000667
		-0.000250	-0.00108	-0.00225	-0.00140	-0.00142	-0.00225
		0.00242	0.00117	0.00131	0.00150	0.00117	0.000667

In each cell, the first number corresponds to the median of the prediction difference, while the second and third numbers refer to the 25% and 75% quantiles, respectively.

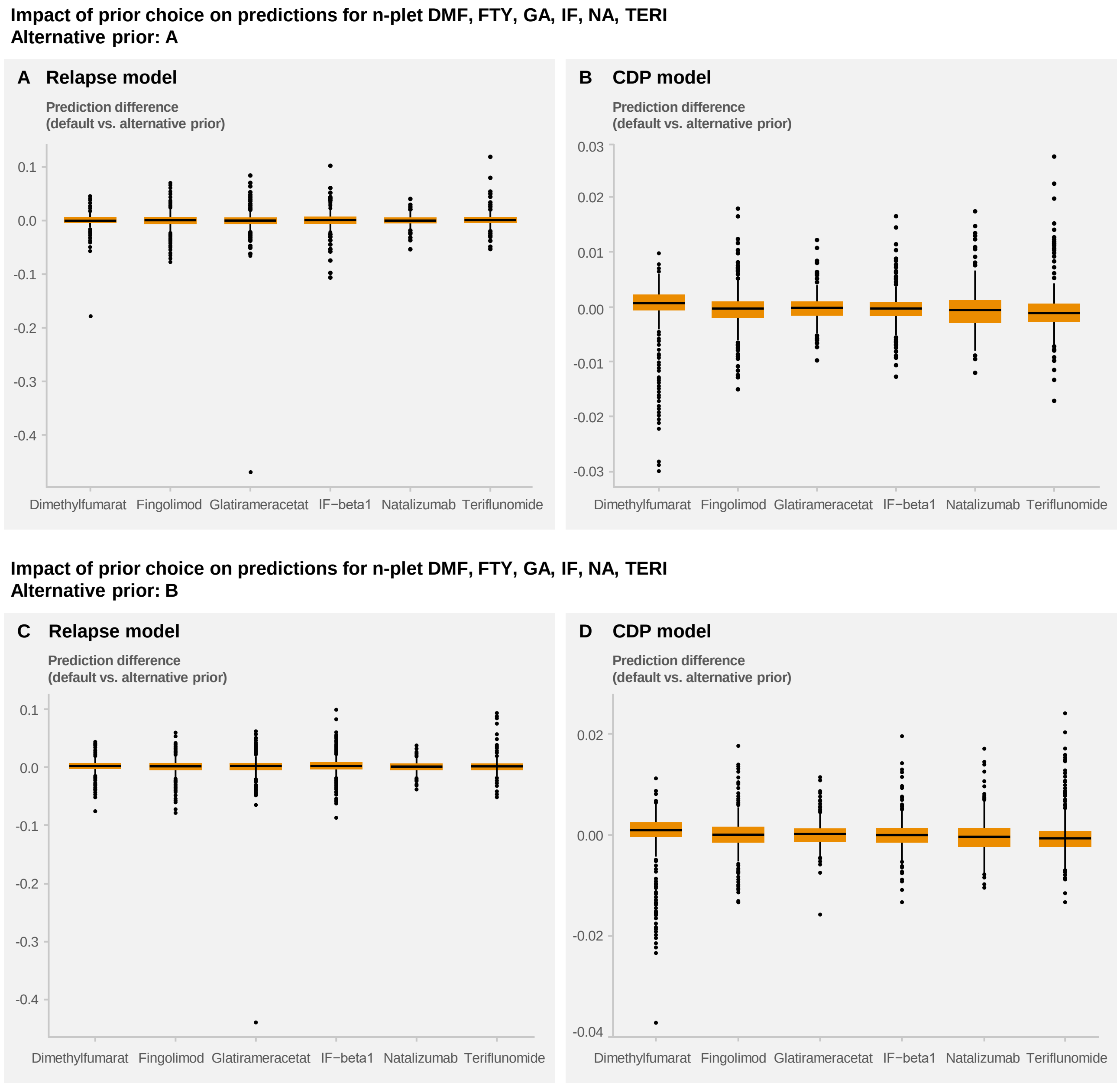

Figure S5.1: Impact of a different choice of the priors on the predictions, as obtained for the relapse model (left) and for the CDP model (right) when using the priors of scenario A (top) or the priors of scenario B (bottom). The boxplots show the change in the predictions induced by the alternative priors compared with the default priors, for each DMT under consideration.

Impact of prior choice on model coefficients
Differences between posterior medians of model coefficients (default vs. alternative prior)
Relapse model
Alternative prior: A

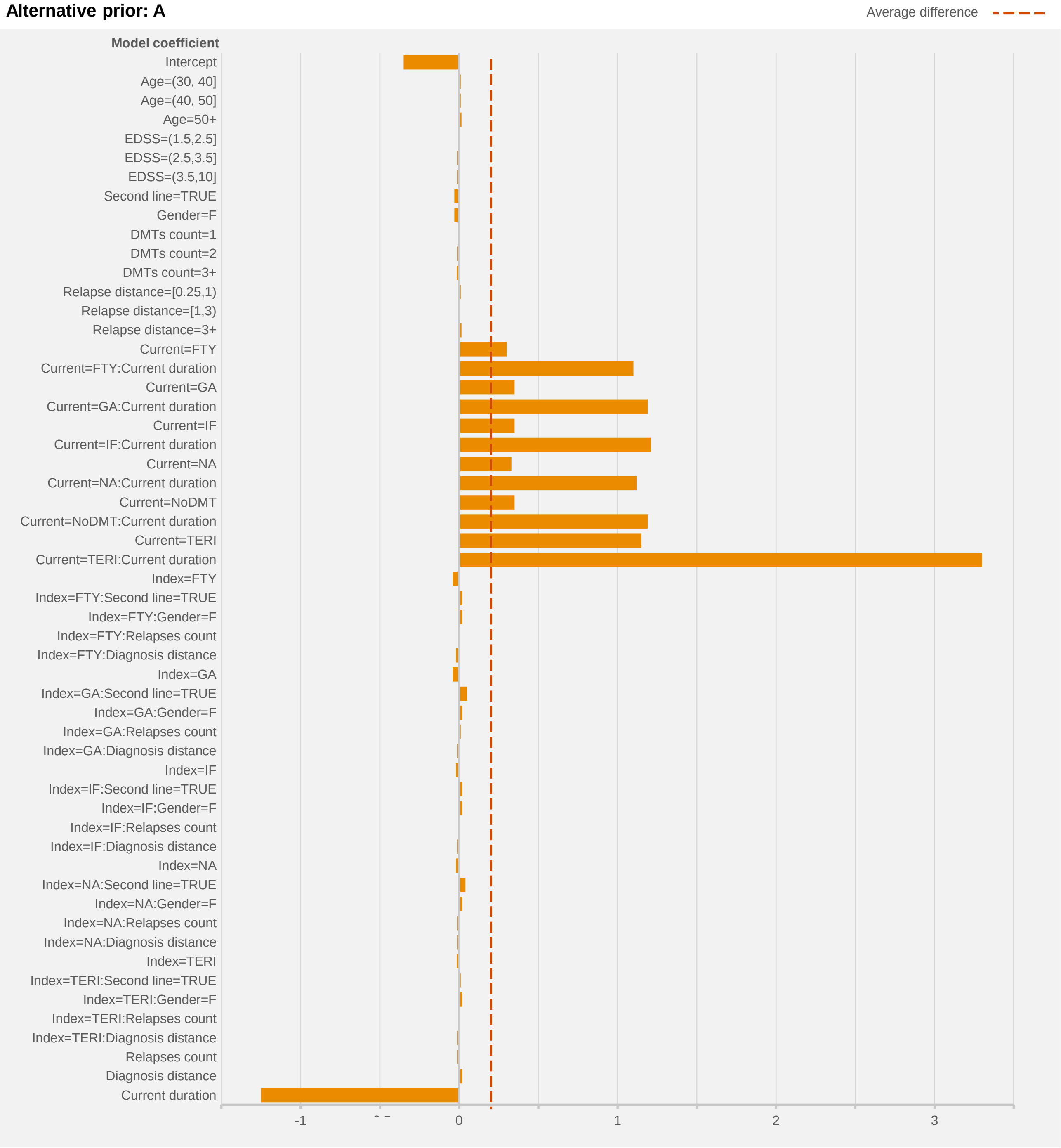

Figure S5.2: Change in the posterior medians of the fixed-effect parameters induced by the alternative priors compared with the default priors, as obtained for the relapse model when using the priors of scenario A. The predictor associated with the parameter is shown on the vertical axis, while the difference between the medians is shown on the horizontal axis. The dashed red line shows the average difference between medians.

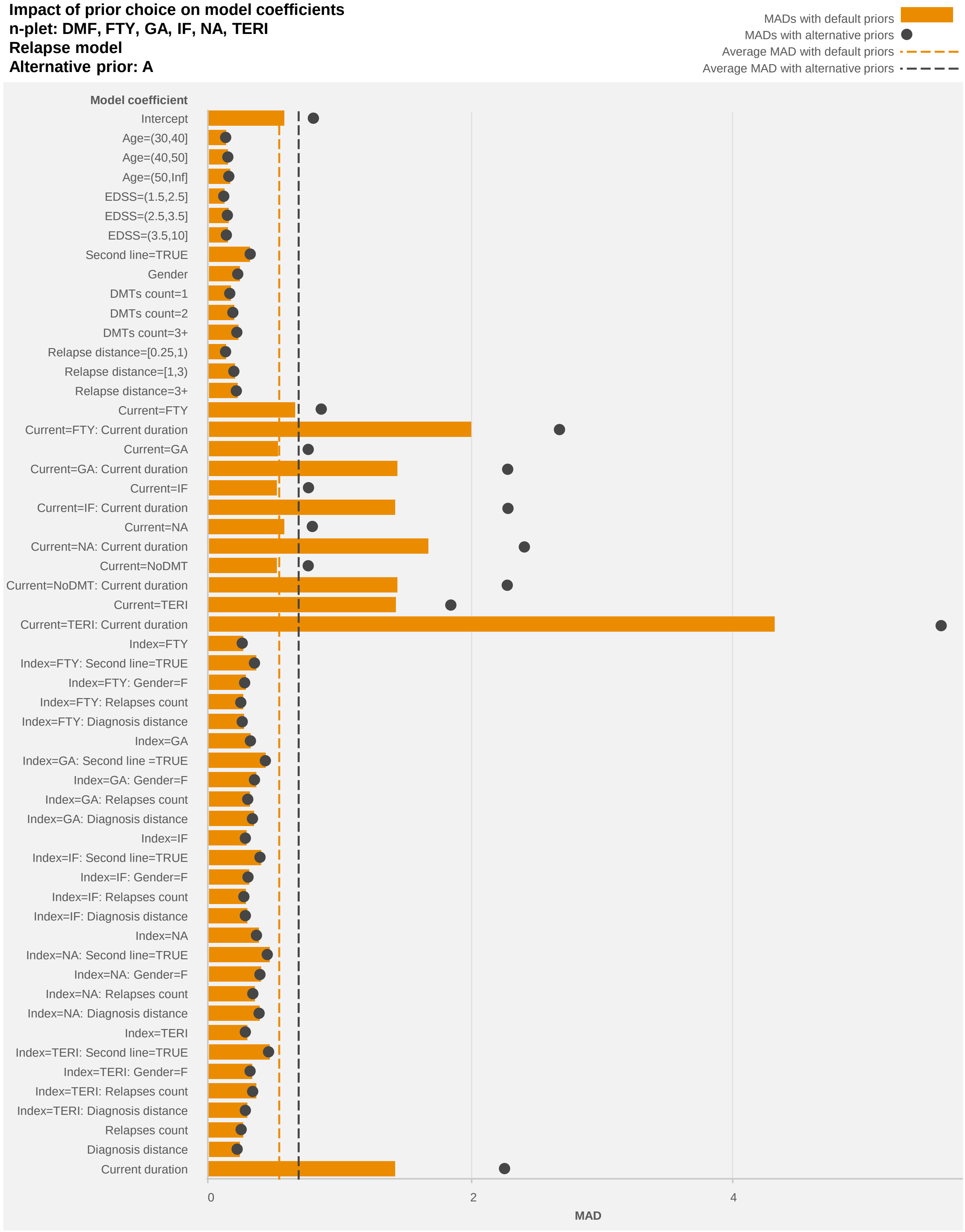

Figure S5.3: Posterior MADs of the fixed-effect parameters, as obtained for the relapse model when using the default priors (orange bars) and when using the priors of scenario A (gray dots). The predictor associated with the parameter is shown on the vertical axis, while the MAD is shown on the horizontal axis. The dashed lines show the average MAD under the default priors (orange) and under the priors of scenario A (gray).

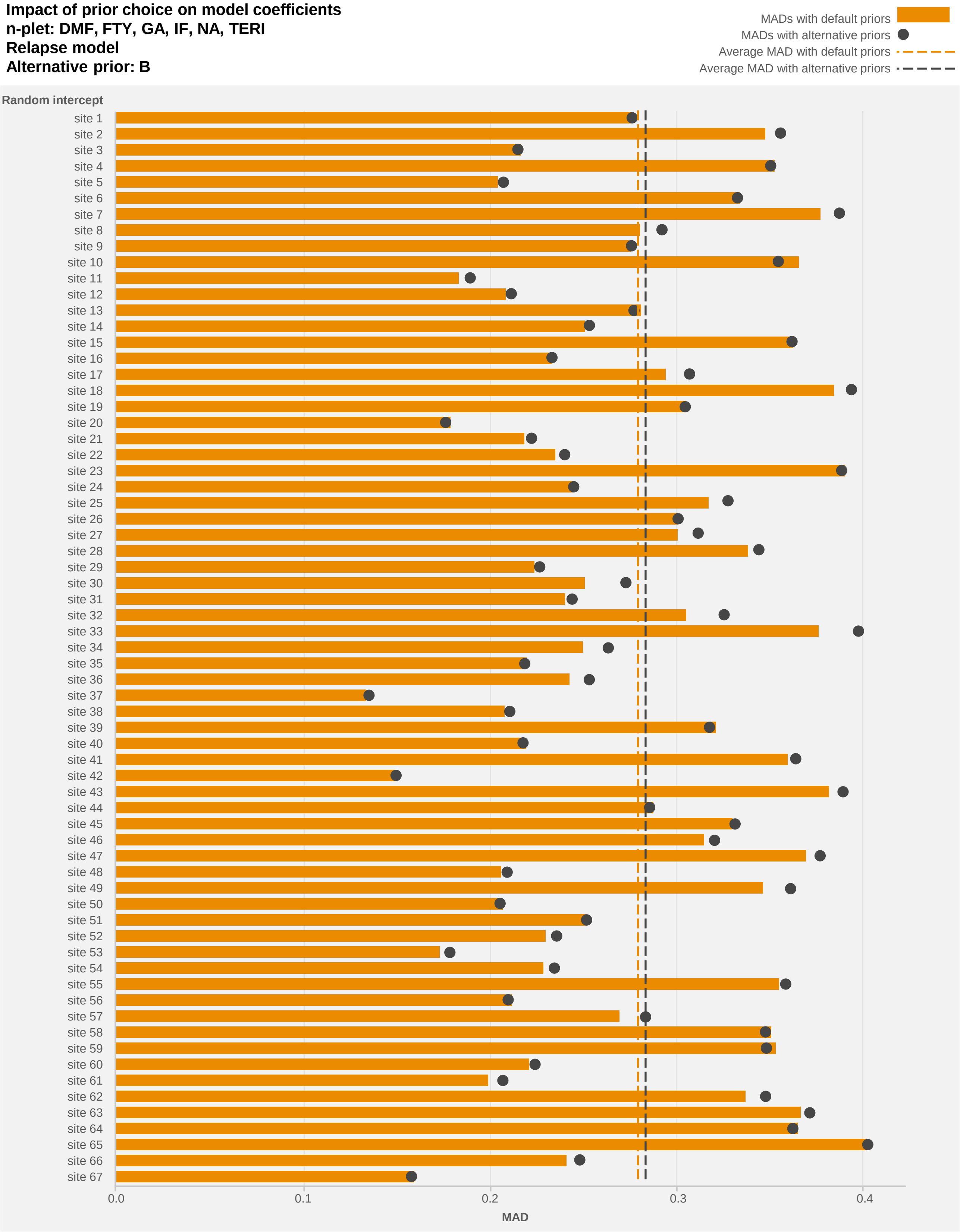

Figure S5.4: MADs of the random intercepts, as obtained for the relapse model when using the default priors (orange bars) and when using the priors of scenario B (gray dots). The clinical site associated with the random intercept is shown on the vertical axis, while the MAD is shown on the horizontal axis. The dashed lines show the average MAD under the default priors (orange) and under the priors of scenario B (gray).

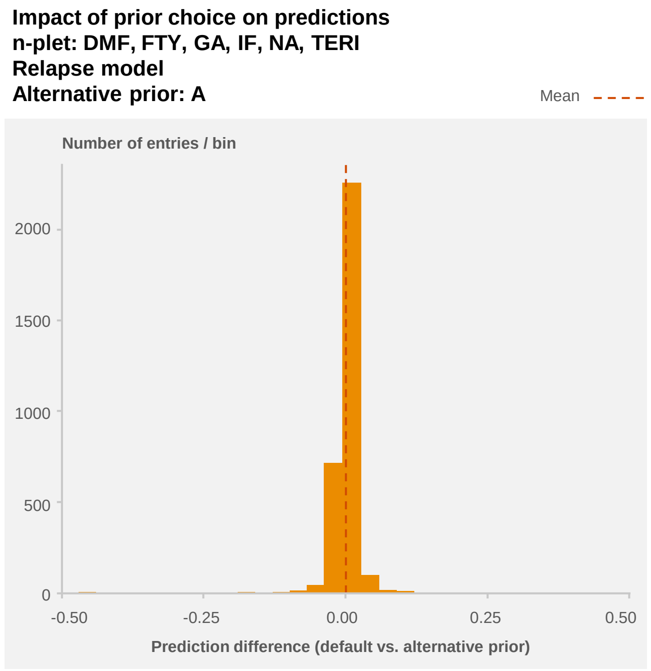

Figure S5.5: Histogram of the prediction difference between default and alternative priors, as obtained for the relapse model when using the priors of scenario A. The dashed line shows the mean of the distribution.

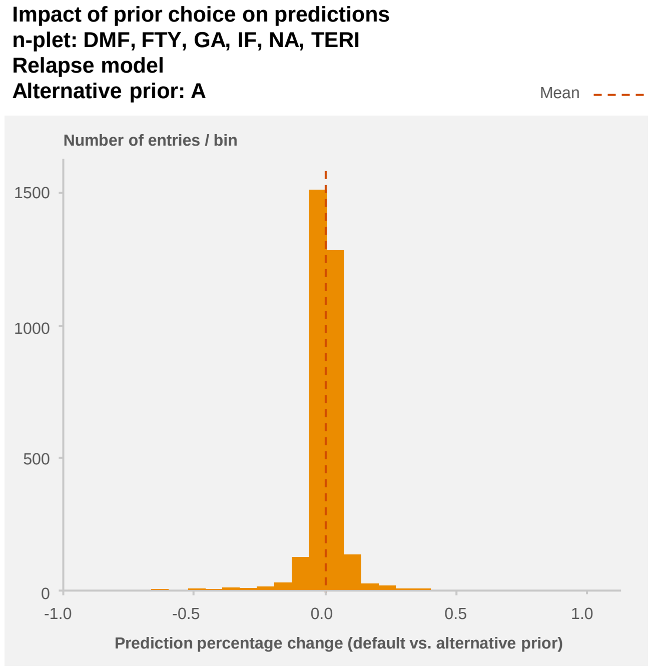

Figure S5.6: Histogram of the prediction percentage change between default and alternative priors, as obtained for the relapse model when using the priors of scenario A. The dashed line shows the mean of the distribution.

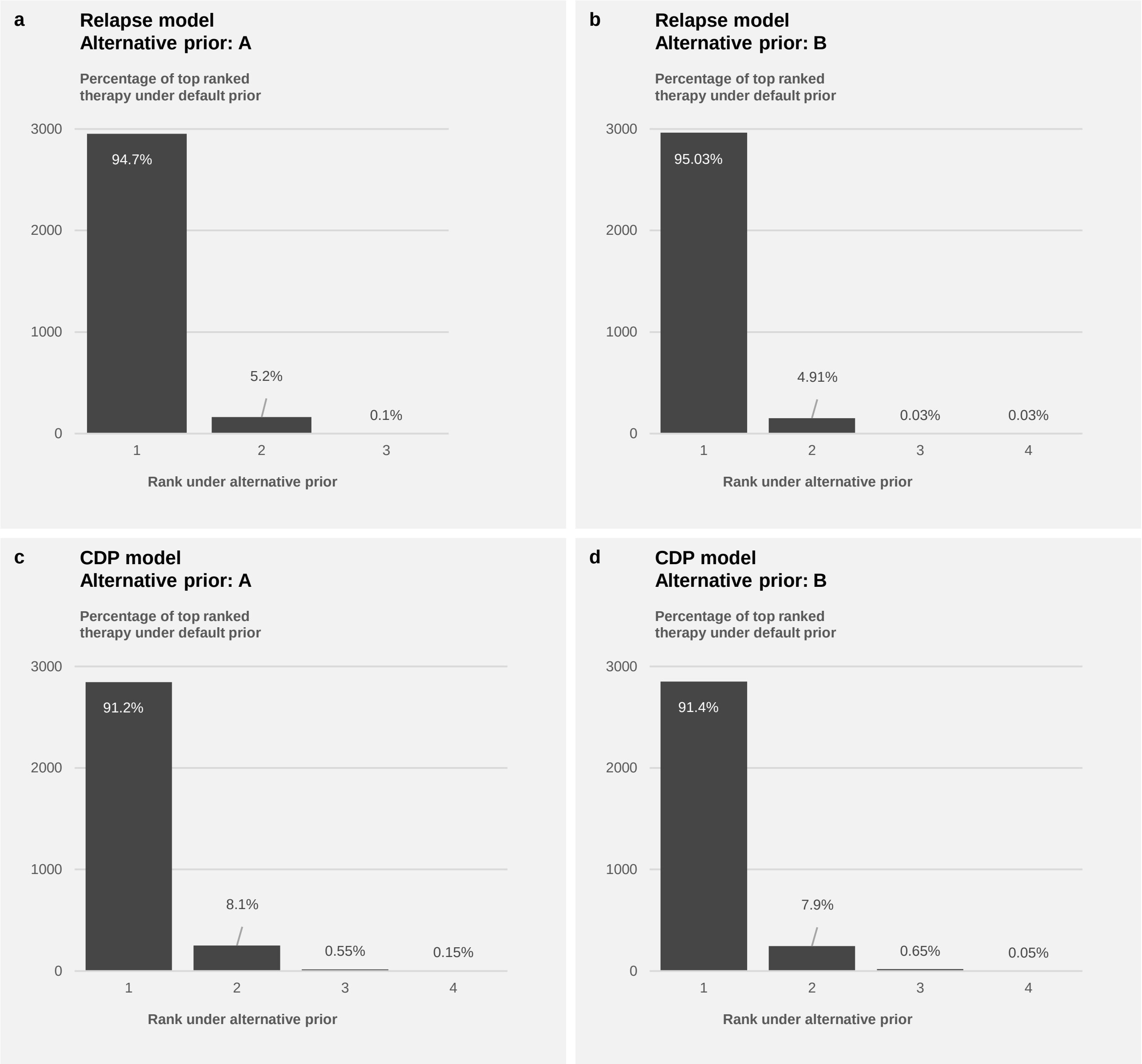

Figure S5.7: Changes in the therapy ranking induced by the adoption of alternative priors. In particular, percentage of transitions of the highest-ranked therapy under the default priors to highest-ranked, second-ranked and third-ranked therapy under the alternative priors, in percentage of the total number of therapy cycles under consideration
