# Supplementary material for: Framework for personalized prediction of treatment response in relapsing remitting multiple sclerosis
Source: BMC Med Res Methodol. 2020 Feb 7;20:24. doi: 10.1186/s12874-020-0906-6 (PMC7006411; doi:10.1186/s12874-020-0906-6)
Supplement: Supplementary file 6 — Additional file 6. “Model robustness against differences in patient characteristics”: Supplementary methods and results. [file 12874_2020_906_MOESM6_ESM.pdf]

Additional file 6: Model robustness against differences in patient characteristics

1. Methods

The model performance might be affected by an imbalance of the characteristics of the patient population across treatment arms, that is, across observed therapies. In order to ensure that differences in patient characteristics across treatment arms do not impact the model performance in a significant way, both predictive models were fitted to three different subsets of the data set: propensity score matched[1], unmatched without stratification, and unmatched and stratified in each treatment arm (as sample size is considerably different). Due to limited sample size all six therapies could not be matched simultaneously. Therefore, this strategy was adopted for three clinically relevant combinations of therapies, which represent frequent clinical decision scenarios in RRMS: (1) Dimethylfumarat, Fingolimod and Natalizumab representing cases with therapy escalation, (2) Dimethylfumarat, IF-beta and Teriflunomide representing decisions between oral vs. injectable DMTs, and (3) Glatirameracetat, IF-beta and Teriflunomide representing long established injectable DMTs with a recently available new oral DMT. Statistical measures were computed using 10-fold cross-validation and compared across different choices of underlying data.

2. Results

As a sensitivity analysis, the validity and accuracy of the prediction of treatment effectiveness was compared in between three clinically relevant therapy triplets based on propensity score matched patient populations. Overall there were initially 1712 (1), 1690 (2) and 1407 (3) patients in each of these therapy triplets, after applying 1:1 propensity score matching or stratification 669 (1), 1068 (2) and 906 (3) patients remained.

The model performance achieved by models fitted on unmatched and matched populations was assessed by two performance measures grouped by the observed therapy triplet. For CDP and relapse models no clear differences or trends are apparent for the relationship between C-Index on matched versus non matched populations (Table S6.1.a). The same statement applies to MSE (Table S6.1.b). In summary, these findings demonstrate robustness of the predictive results independent of differences in the underlying populations as they did not impact model performance w.r.t discrimination or goodness-of-fit.

---

[1] G. Ridgeway, D. McCaffrey, A. Morral, B. A. Griffin and L. Burgette, "Twang: Toolkit for Weighting and Analysis of Nonequivalent Groups," [Online]. Available: https://cran.r-project.org/web/packages/twang/index.html. [Accessed October 2018].

Table S6.1: Sensitivity against population differences in treatment arms

a: C-Index

		Matched and stratified	Unmatched and stratified	Unmatched and unstratified
DMF, FTY, NA	CDP	0.610511182	0.618095376	0.602996789
	Relapse	0.638669119	0.613897492	0.631635056
DMF, IF, TERI	CDP	0.526190414	0.586963469	0.585825044
	Relapse	0.634329049	0.649659666	0.650725273
GA, IF, TERI	CDP	0.523199259	0.572811254	0.542698119
	Relapse	0.617311364	0.612747928	0.637146573

b: MSE

		Matched and stratified	Unmatched and stratified	Unmatched and unstratified
DMF, FTY, NA	CDP	0.130897427	0.123619044	0.12427246
	Relapse	0.966492415	1.209850317	0.755265382
DMF, IF, TERI	CDP	0.12126664	0.124823231	0.119311017
	Relapse	0.672841948	0.840075357	0.653216243
GA, IF, TERI	CDP	0.12708204	0.126568431	0.127325307
	Relapse	0.945673447	1.766286881	0.842375502

C-index and MSE based on out-of-sample evaluation by 10-fold cross-validation. Predictive models were fitted to matched and unmatched data. Their out-of-sample performance is shown for the two performance measures C-Index (a) and MSE (b).
