# Supplementary material for: Framework for personalized prediction of treatment response in relapsing remitting multiple sclerosis
Source: BMC Med Res Methodol. 2020 Feb 7;20:24. doi: 10.1186/s12874-020-0906-6 (PMC7006411; doi:10.1186/s12874-020-0906-6)
Supplement: Supplementary file 7 — Additional file 7. “Model robustness against different training set size”: Supplementary methods and results. [file 12874_2020_906_MOESM7_ESM.pdf]

Additional file 7: Model robustness against different training set size

1. Methods

As the volume of data in the NTD database is steadily growing with close to 15% annual growth rate, models fitted to future database extracts are expected to be more stable. The sensitivity of the model to training sample size was evaluated by fitting a series of both predictive models on an increasing fraction of the whole training data, sampled randomly. This is done for a sample size corresponding to 50%, 70%, and 90% of the whole training data. For every 10-fold cross-validation iteration, pairwise differences between ten coefficient sets coming from ten folds were calculated and collected in order to assess if and how much they stabilize for an increasing sample size.

2. Results

Initial work on the models suggested that the sample size might have an effect on model parameters and overall performance. Pairwise differences between model coefficients estimated in 10-fold cross-validation are compared for models build on progressively smaller subsamples of the full training set.

Figure S7.1 (a) shows that model coefficients become very similar between predictive relapse models trained on the full population and subsamples consisting of 70% or 90% of the data. The same does not hold for the predictive CDP models (**Figure S7.1** (b)), where change in pairwise difference of coefficients is still indicated to be significant when comparing models trained on 100% and 90% of the full data.

These findings confirm an important effect of sample size on the predictive models. The fact that 90% of data results in similar predictive models as models trained on the full data set suggests that the current data set size is already sufficient to get stable model coefficients for the relapse model, while the CDP model might benefit from additional data.

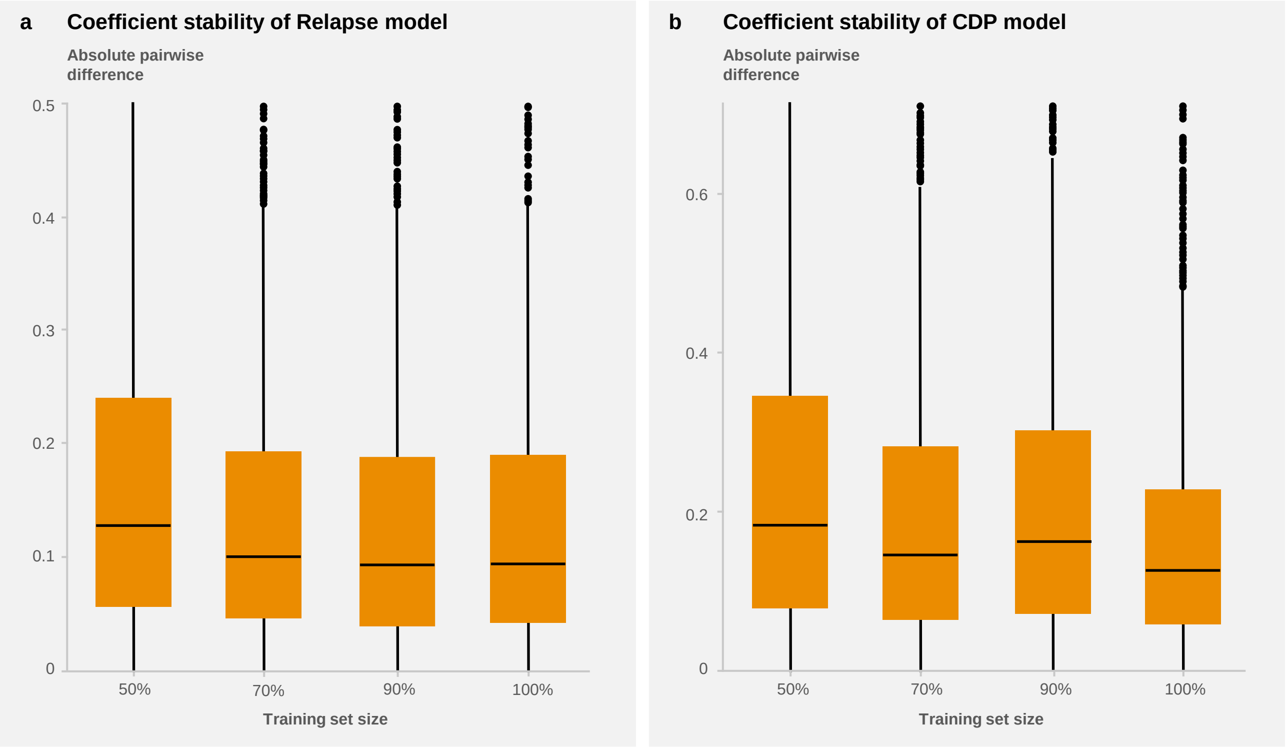

Figure S7.1: Absolute pairwise differences of coefficients of the predictive relapse model fit (a) and CDP model fit (b). For each model parameter a set of 10 coefficients was derived during 10-fold cross-validation, and pairwise differences were computed within this set. 10-fold cross-validation was conducted based on increasing training set size to evaluate if and when coefficients stabilize with increasing sample size. This is the case for the relapse model (a) where coefficients stabilize after 70% of data have been seen, whereas the CDP model still shows higher and less stable pairwise differences.
