# Supplementary material for: Framework for personalized prediction of treatment response in relapsing remitting multiple sclerosis
Source: BMC Med Res Methodol. 2020 Feb 7;20:24. doi: 10.1186/s12874-020-0906-6 (PMC7006411; doi:10.1186/s12874-020-0906-6)
Supplement: Supplementary file 8 — Additional file 8. “Comparison of predicted treatment effectiveness”: Supplementary tables. [file 12874_2020_906_MOESM8_ESM.pdf]

Additional file 8: Comparison of predicted treatment effectiveness

Table S8.1: Sample sizes for highest ranked therapy

Clinical Outcome	Number of Relapses		Occurrence of CDP	
DMT*	Sample Size for DMT* Taken	Sample Size when DMT* Not Taken	Sample Size DMT* Taken	Sample Size DMT* Not Taken
Dimethylfumarat	134	307	863	306
Fingolimod	128	112	87	135
Glatirameracetat	-	1	45	238
IF-beta1	2	6	3	14
Natalizumab	234	2182	132	1101
Teriflunomide	-	13	16	179

The number of patients for each highest ranked therapy (DMT*) is listed based on their adherence. Sample sizes are listed based on the mean posterior number of relapses, and based on the mean posterior occurrence of a CDP.

Table S8.2: Survey-weighted GLM for the predictive relapse model

GLM model outputs (svyglm.nb)			
	Coefficients	SE	p-value
Dimethylfumarat: intercept	-1.0705	0.1385	<0.001
Dimethylfumarat: slope	-0.6918	0.2892	0.0167
Fingolimod: intercept	-0.8143	0.1711	<0.001
Fingolimod: slope	0.0423	0.24	0.86
Natalizumab: intercept	-0.7421	0.0418	<0.001
Natalizumab: slope	-0.4376	0.1873	0.0195

SE: Standard error of the coefficients. Model outputs of the survey-weighted negative binomial GLM svyglm.nb, applied to the observed number of relapses. The survey-weighted GLM was fitted to each therapy in turn. Group membership (subject who received the therapy ranked highest by the predictive model and those who received another therapy) was encoded by an indicator variable and derived from the predictive relapse model.

Table S8.3: Survey-weighted GLM for the predictive CDP model

GLM model outputs (svyglm)			
	Coefficients	SE	p-value
Dimethylfumarat: intercept	-2.36254	0.09642	4.18E-107
Dimethylfumarat: slope	-0.53629	0.243424	0.027783622
Fingolimod: intercept	-2.59057	0.290462	2.34E-16
Fingolimod: slope	0.111356	0.422328	0.792290399
Glatirameracetat: intercept	-2.24036	0.174009	4.15E-30
Glatirameracetat: slope	-0.53358	0.569995	0.3500183
Natalizumab: intercept	-2.27615	0.083491	9.42E-129
Natalizumab: slope	-0.40208	0.482948	0.405249923
Teriflunomide: intercept	-2.37646	0.223784	3.24E-21
Teriflunomide: slope	-0.4317	1.249648	0.730108299

SE: Standard error of the coefficients. Model outputs of the survey-weighted quasi-binomial GLM svyglm, applied to the observed occurrences of CDP. The survey-weighted GLM was fitted to each therapy in turn. Group membership (subject who received the therapy ranked highest by the predictive model and those who did not) was encoded by an indicator variable and derived from the predictive CDP model.
