# Supplementary material for: Framework for personalized prediction of treatment response in relapsing remitting multiple sclerosis
Source: BMC Med Res Methodol. 2020 Feb 7;20:24. doi: 10.1186/s12874-020-0906-6 (PMC7006411; doi:10.1186/s12874-020-0906-6)
Supplement: Supplementary file 9 — Additional file 9. “Clinical site effect”: Supplementary figures. [file 12874_2020_906_MOESM9_ESM.pdf]

Additional file 9: Clinical site effect

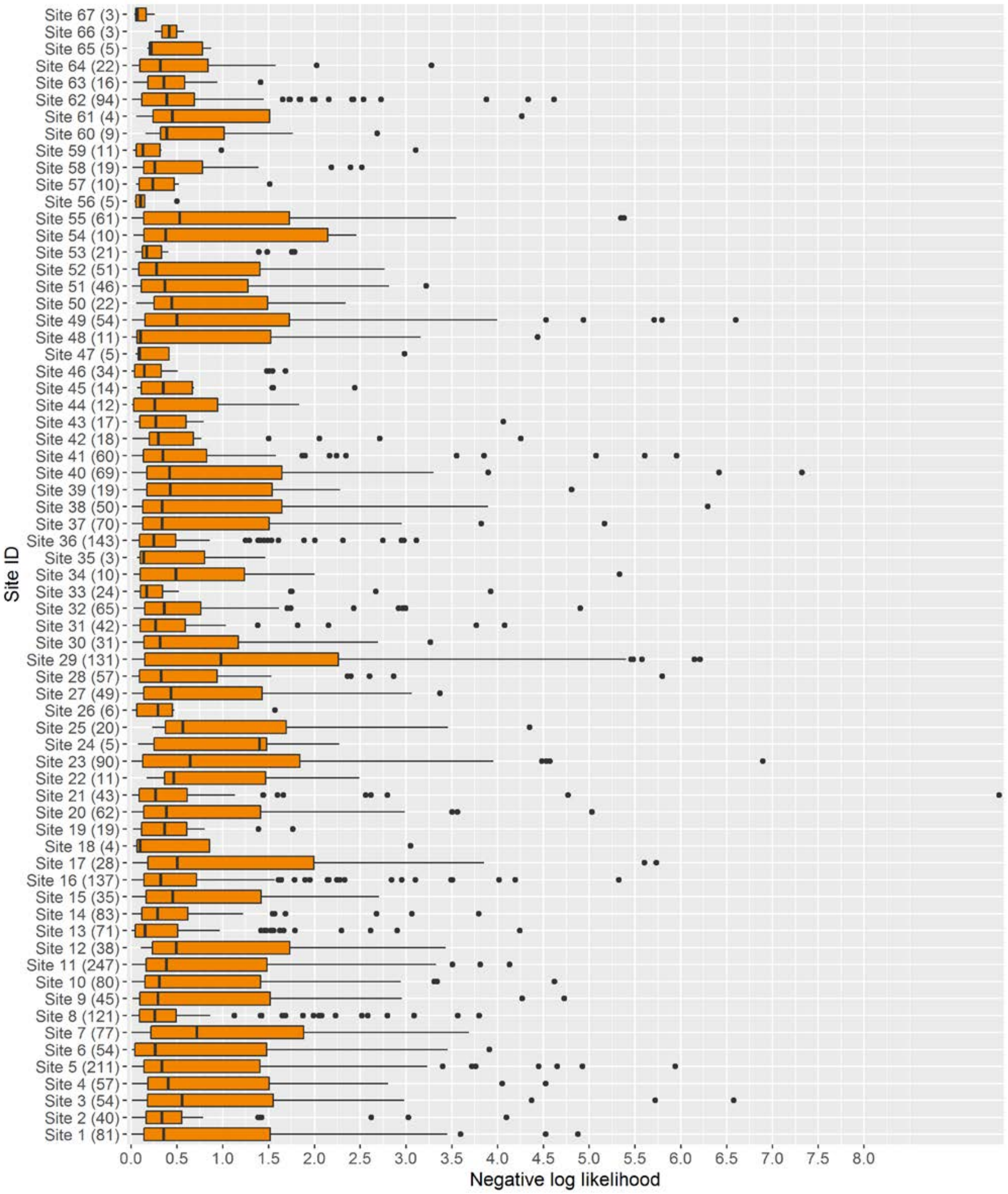

Figure S9.1 Performance by clinical site of the relapse model. Prediction performance of the relapse model for all clinical sites, measured by the negative log-likelihood. For every site, a boxplot of negative log-likelihoods for the patients being part of the site is shown. Numbers in brackets indicate the number of patients for a given clinical site.

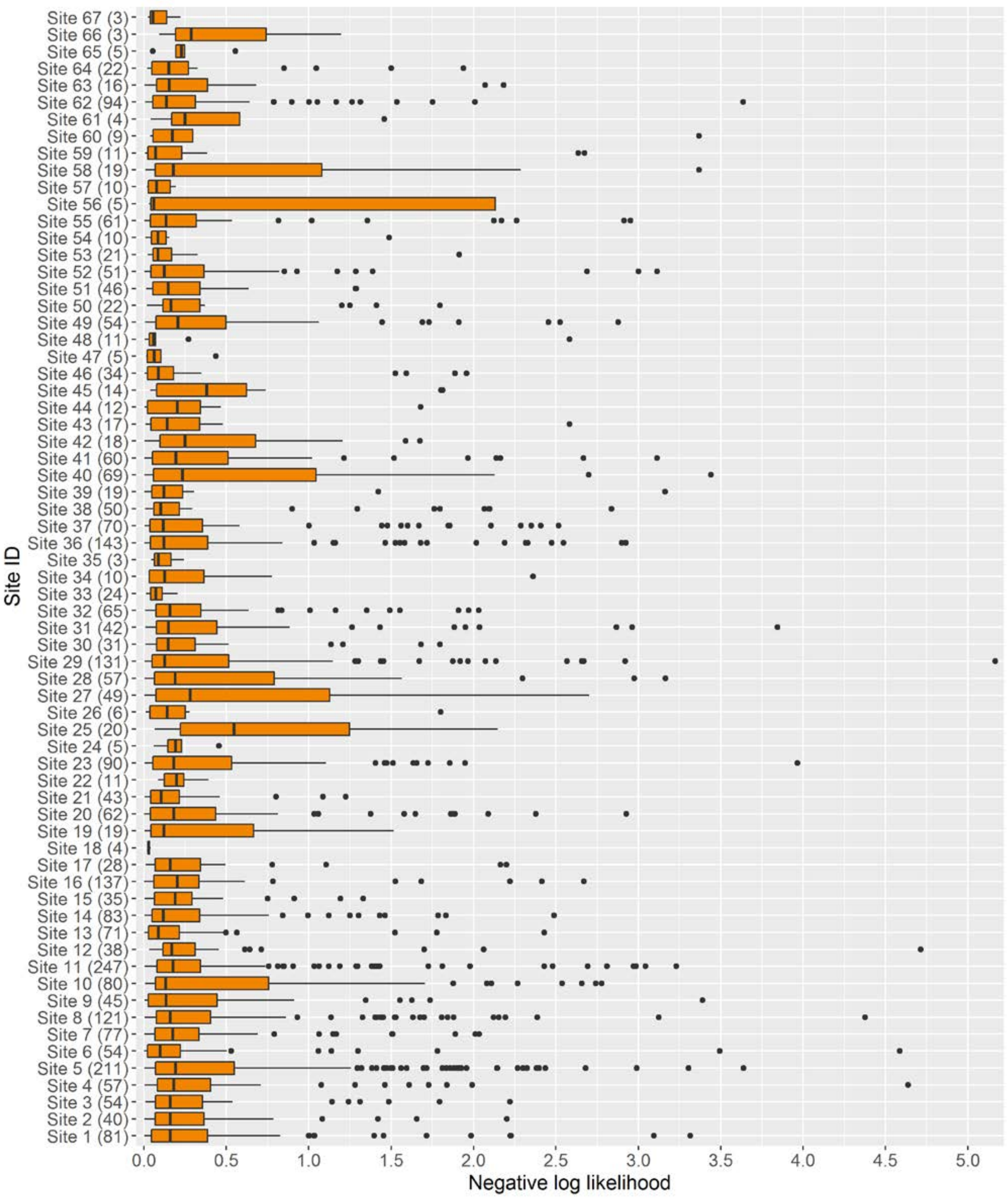

Figure S9.2 Performance by clinical site of the CDP model. Prediction performance of the CDP model for all clinical sites, measured by the negative log-likelihood. For every site, a boxplot of negative log-likelihoods for the patients being part of the site is shown. Numbers in brackets indicate the number of patients for a given clinical site.
